# Local and landscape factors affect sunflower pollination in a Mediterranean agroecosystem

**DOI:** 10.1371/journal.pone.0203990

**Published:** 2018-09-27

**Authors:** Agustín M. Bartual, Gionata Bocci, Simone Marini, Anna Camilla Moonen

**Affiliations:** Institute of Life Sciences, Scuola Superiore Sant’Anna, Pisa, Italy; Philipps-Universitat Marburg Fachbereich Biologie, GERMANY

## Abstract

In Europe, the surface devoted to sunflower cultivation has expanded by ∼ 26% from 2006 to 2016. Theoretically, this implies an increasing demand for pollinators, while at the same time, scientific reports claim that pollinator communities worldwide are threatened by multiple stressors such as agrochemicals, the loss of suitable habitats and habitat fragmentation. However, the question that arises is whether insect pollination is still relevant for modern sunflower varieties that are often highly self-fertile. Following recent studies which demonstrate that surrounding land use composition may affect ecosystem service provisioning in cropped fields, this study aims at re-examining the pollination status of sunflower while disentangling the effects of local and landscape variables on sunflower seed set and oil content in Central Italy. Commercial cultivars, regardless of their degree of self-fertility, showed increased seed set and oil content when receiving adequate amounts of cross-pollination; oil composition, though, was not affected by cross-pollination events. Honey bees accounted for the vast majority of pollinators ensuring an overall adequate pollination. Sunflower seed set was higher in fields surrounded by landscapes containing a greater abundance of beehives, early flowering crops, urban areas and woody linear elements; conversely, seed set was lower where herbaceous semi-natural habitats dominated the surrounding landscape. This information is necessary for a science-based planning of agricultural policies and shows that, despite the adoption of self-fertile cultivars, sunflower still benefits from insect pollination and land use planning may affect crop productivity.

## Introduction

One of the main challenges when developing innovative and sustainable cropping systems is to maximise ecosystem service provisioning while securing food production for the future. Crop pollination is a frequently discussed issue in this regard, but the complexity of crop-pollinator interactions does not allow for an unequivocal interpretation of results and simple management guidelines for all pollinator-dependent crops. Analysing data from 1961 to 2008, Garibaldi et al. [1] showed that average yield increased while yield variability decreased with decreasing dependence on pollinators. In the meantime, crop cultivar selection has greatly modified crop characteristics towards higher levels of self-fertility and higher quality products for human consumption, which may alter the relationship between crops and pollinators and consequently the effect of cross-pollination on yield quantity and quality.

Assuming that also modern varieties of dependent crops rely on insect pollination, the alarming declines in managed honey bees [2, 3], bumble bees [4] and other wild bees [5–7] raises the need to examine the delivery of pollination services [8], as well as to determine whether crop yield is already being compromised by pollination deficits [9]. Notwithstanding an increase in the global stock of commercial honey bee colonies by ∼ 45% from 1961 to 2008 [10], consistent declines in colony numbers were simultaneously registered in central European countries [3] and in the U.S.A. [11]. The main causes for worldwide pollinator declines are habitat loss or fragmentation, land use changes and modern agricultural practices [6]. These declines may result in global risks of food security and ecosystem stability [6, 12] (nonetheless, some controversy has arisen around this issue, e.g. [13, 14]) and have caused concern among scientists, policy makers and the general public about possible imbalances between the pollination levels that are needed and those actually supplied [15]. To prevent an ecologically and agronomically risky situation, several strategies have been proposed, such as: (i) diversification of the available crop pollinators via the protection or domestication of other bee species [16], (ii) conservation or incorporation of semi-natural habitat patches (hereafter SNH) into the agricultural matrix [17–19], or (iii) reduction in pesticide use [17, 20].

Proximity of SNH patches is usually a factor which positively affects visitation rates and crop yield [18]. Furthermore, some studies suggested that SNH patches in the landscape may buffer negative effects of intensive agriculture on pollinators [21, 22]. However, SNHs have often been related to crop pollination independently of their shape (linear or areal patches) and cover typology (grassy or woody patches), which are known to be two key factors in shaping vegetation communities [23] and may therefore determine whether the SNH will act as a source or a sink for beneficial insects, including crop pollinators [24, 25].

Sunflower (*Helianthus annuus* L.) is an economically important oilseed crop in the European Union. From 2006 to 2016 its cultivated area increased by ∼ 26% [26] with an average production of 7.6 million tonnes per year. Since this crop depends, at least partially, on insect mediated pollination, the role of managed honey bees (*Apis mellifera* L.) and wild bees has long been investigated [27–32]. The positive yield response of sunflower to honey bee visits has been demonstrated by many authors [33, 34], although others have noted that their behaviour is not adequate to guarantee a sufficient cross-pollination on hybrid cultivars [35]. These contrasting results suggest that, despite not being the most efficient pollinators, they may be effective enough given their high numbers. On the contrary, the role of wild bees in sustaining sunflower pollination is less clear, since it appears more context dependent as a result of the wide diversity of wild bee species with, very often, important behavioural differences. In the native area of sunflower, North America, many different wild bees visit commercial sunflower [28, 36, 37], while wild *Helianthus* species are visited by up to 400 native wild bee species, including many oligolectic bees [38, 39]. In addition, wild pollinators may indirectly increase sunflower pollination by affecting honey bee behaviour during sunflower visits [36, 40], even if this hypothesis is not supported by data in other regions [41].

Sunflower has historically been an important crop in Central Italy, so that during the 1970s several studies were performed to identify its key pollinators. In these studies, the most abundant visitors were honey bees, which is not surprising since the activity of bee-keepers has also been traditionally linked to this crop for honey production [42, 43], whereas the most abundant wild bees belonged to the *Bombus* and *Halictus* genera [44, 45]. In those days, sunflower cultivars distributed in the study area were highly dependent on cross-pollination, and self-pollinated heads produced only between 2 and 16% of filled achenes depending on the variety [46]. However, following the introduction of new sunflower cultivars which are often highly self-fertile, the relevance of insect pollination in sunflower may have decreased. On the other hand, since the market prefers high-quality oil for human consumption, high oleic sunflower varieties have been recently introduced in the area. As a result, the request for high-quality oil could stress the importance of pollinating agents, which may increase the sunflower oil quality, as demonstrated for other pollinator-dependent crops [47–49]. To the best of our knowledge, previous studies have only focused on the influence of genetic inheritance or abiotic factors on fatty acid composition of sunflower oil [50, 51], and have not tested if cross-pollination may affect the percentage of oleic acid.

In this context, the present work aimed at, firstly, re-examining the status of sunflower pollination in Central Italy by characterising the pollinator assemblage and its influence on seed set and oil content of modern sunflower varieties; and secondly, following recent studies that demonstrate the role of land use composition on ecosystem service provisioning [52–54], it aimed at disentangling the effect of local and landscape land use types on pollination service delivery.

The following hypotheses were tested: (i) despite the high level of self-fertility in modern sunflower varieties, insect pollination is still able to boost sunflower seed set and oil content, and may also affect oil composition; (ii) actual pollination levels do not result in diminished yields in the study area; (iii) crop pollination is affected by land use types in the surrounding landscape and by the density of beehives.

## Material & methods

### Study area and experimental design

This study was conducted during two consecutive seasons (2014 and 2015) in the region of Pisa, Italy (43° 39’ N, 10° 28’ E, see Fig A in S1 Appendix), an alluvial plain characterized by medium-scale patches of arable crops (mean patch size ± S.D.: 2.0 ± 2.2 ha) and a Mediterranean climate with dry and hot summers and cool and rainy winters (mean annual temperature 14.8°C, August is the hottest month with 24.6°C; mean annual precipitation 866 mm, November is the rainiest month with 137 mm). Elevation ranged from 4 to 75 m a.s.l. Focal fields were selected in a matrix of Mediterranean wood and shrubland, channel banks (often hosting trees and shrubs), herbaceous patches and grassy tracks, with urban agglomerations also present in the area (see Tables D and E in S1 Appendix for further information). In this area, sunflower is not irrigated, being an occasional crop in a wheat-based crop rotation where various summer crops follow winter wheat. Often this rotation is broken by a 3 or 4 year alfalfa crop. Bee-keepers commonly place their hives near fields of mass-flowering crops such as sunflower and alfalfa. The maximum hive density in the area is regulated by regional Tuscan law: nomadic apiaries with 50 or more beehives need to be at least 200 m apart.

**Fig 1:**
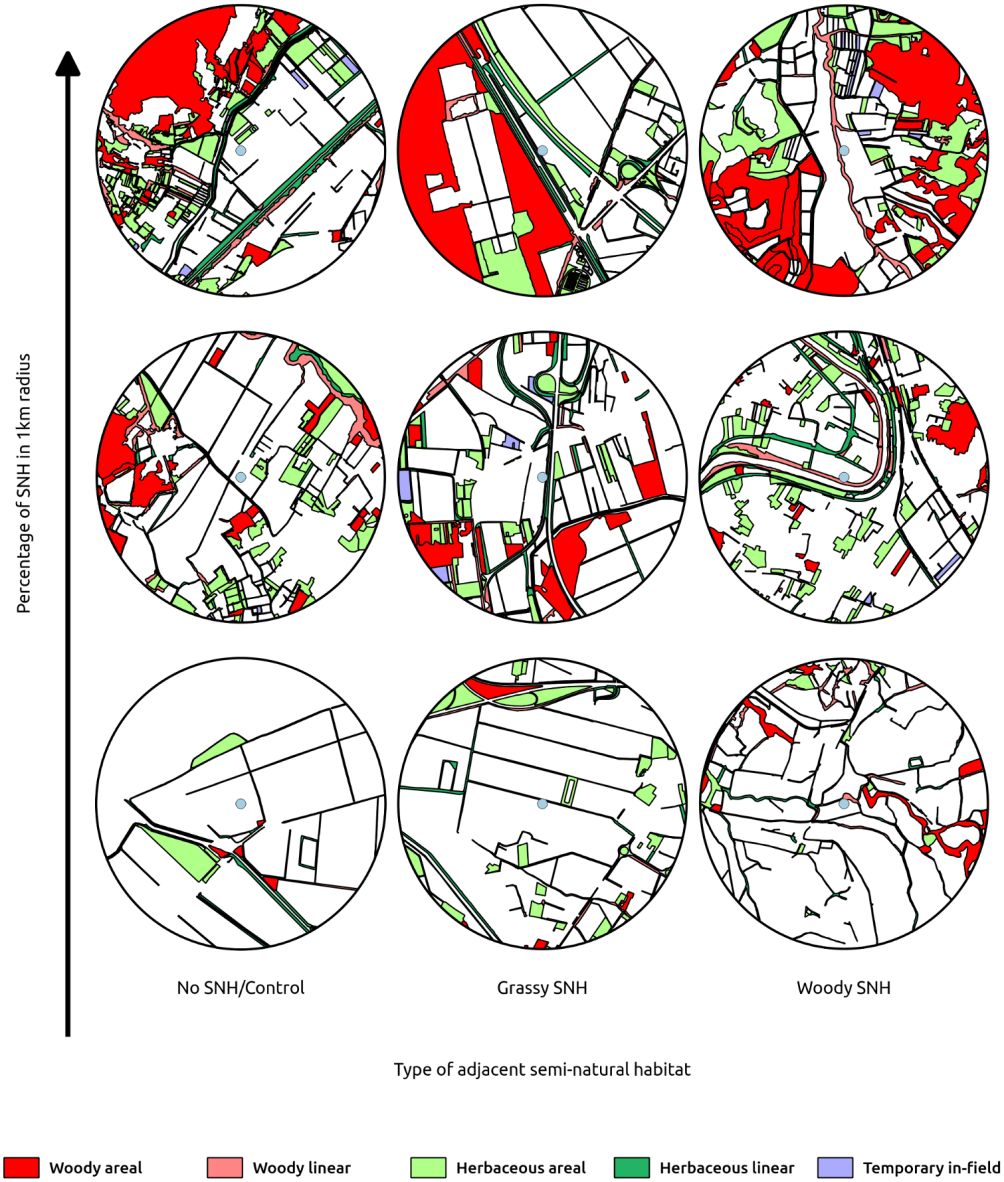
**Digitalized maps of a subset of the sampled landscape sectors** are here shown to illustrate the adopted design: for each of the three focal side typologies (no SNH, grassy SNH and woody SNH; columns in the figure) fields were chosen so that a gradient of landscape complexity (percentage of SNHs) was represented in a 1 km-radius buffer, ranging from simplified ones (bottom row) to more complex ones (top row).

The methodology described below has been developed as part of the QuESSA EU FP7 project [55]. During the first season, 18 commercial sunflower fields (hereafter ‘focal fields’) were chosen in order to obtain a balanced design and avoid confounding effects between local and landscape variables in terms of (i) typology of their adjacent semi-natural element (located at one of the field sides, named the ‘focal side’), and (ii) complexity of the surrounding landscape. The 6 fields per focal side typology [‘herbaceous linear SNH’ (n = 6), ‘woody linear SNH’ (n = 6) and ‘no SNH/other crop’ (n = 6)] were selected to cover a broad range of landscape complexity (considering landscape complexity as the percentage of SNHs in a landscape sector of 1 km radius around each focal field, see Fig 1). The selected adjacent elements were classified in accordance to Table D in S1 Appendix (‘herbaceous linear SNH’: any type of linear element (1.5 to 25 m wide) with less than 30% tree/shrub canopy cover; ‘woody linear SNH’: any type of linear element (1.5 to 25 m wide) with more than 30% tree/shrub canopy cover; and ‘no SNH/other crop’: another crop, either perennial or annual, adjacent to the focal field). Each focal field was planted with one of the common cultivars used for oil production in the study area and managed by farmers according to their standard agronomic practices (see Tables A-C in S1 Appendix for details about sunflower varieties and focal field management; selected fields were either organic or conventional). Focal fields had an average size of 7.14 ha with a SD of 5.19 ha. The minimum and the average distances between fields were respectively 1.95 and 17.28 km for 2014 and 1.48 and 18.51 km for 2015. Overlapping among the selected landscape sectors was negligible, with only two sectors having a 0.5% areal overlap.

In order to test for a distance effect in insect visitation and seed set within each focal field, especially for less mobile small wild bees as reported by Saez et al. [32], samples were taken at four distances (2, 16, 30, 44 m) from the focal side, ensuring that the distance to the other (non focal) margins was never less than 55 m. In this way, in case other SNHs were surrounding the field, they were at least 1.25 times further than the focal SNH. At each distance, 12 sunflower heads were selected: eight were left open for natural cross-pollination (‘Open pollination’), two were isolated from any pollinator using fine mesh fibreglass nets (‘Pollinator-excluded’), and the remaining two were subjected to supplemented hand pollination (‘Pollen supplemented’) (further information in Section B in S1 Appendix; see our Youris video https://youtu.be/H1Yrr-gkMEQ?t=1m55s). These three treatments were fundamental for the estimation of the cross-pollination dependence, the pollination deficit and the actual level of pollination. The experiment was repeated in 2015 with modifications to the sampling design according to the results obtained in the first year. Eight fields were selected based on the same criteria as the previous year and in each field only two samples were taken; one at 22 m from the focal side, and one at the field’s furthest point from any border. At each distance 12 plants were subjected to the same three pollination treatments used in the previous year. Since the study was carried out on private land, permission was acquired from the landowners to conduct the study on their land.

### Landscape assessment

All land use types in the 1 km landscape sector around the 18 focal fields of 2014 were mapped based on aerial photographs (resolution: 2m) [56] using QGIS [57] and later ground validated through field inspections. Mapped elements had a minimum area of 75 m^2^; elements such as woodlands, hedgerows, abandoned fields, agricultural grassy tracks and fallows were considered as SNHs (see Table D in S1 Appendix). The area covered by each land use category was calculated and expressed as a proportion of the total area (see Table E in S1 Appendix for descriptive statistics). Negligible or unevenly represented land uses were omitted from the analysis. Omitted land uses covered altogether 17.3% of the overall evaluated area. In order to take into account the potential influence of managed beehives on the pollination service, during the crop blooming season, all apiaries (groups of beehives) within a range of 1.5 km from the focal fields were identified through ground inspection, interviews of bee-keepers, bee-keepers association and the aid of the local health administration unit who monitors the apiaries in the territory. Though honey bees can have much larger foraging ranges [58], we assumed that the pollination service exerted by these beehives would be inversely proportional to the distance. We therefore used the collected data to build a density map of apiaries (‘heatmap’) adopting an Epanechnikov (quadratic) Kernel with a search radius of 1.5 km, weighted by the number of beehives at each location. The value of the heatmap at the centre of the landscape sector was then considered as a proxy for density of apiaries around each focal field.

### Sunflower visitation rates

As soon as half of the sunflower plants in a focal field ranged between the reproductive stages R5.3 and R5.6 (from 30 to 60% of head’s florets open), two observation plots per sampling distance, each one with four plants, were simultaneously observed for 10 minutes by two independent observers recording all visits by pollinators. A visit was registered once the visitor touched the flower reproductive structures, regardless of visit duration and number of florets touched. Visitors were identified in the field. When identification was not possible, the visitor was collected for later identification. All bees (Hymenoptera: Apiformes) were identified at species or morphospecies level; hoverflies and butterflies were classified at family and order level respectively (Syrphidae and Lepidoptera). Subsequently, visitors were grouped into four groups: honey bees (*Apis mellifera* L.), bumble bees (*Bombus* spp), other wild bees (remaining Apiformes) and other pollinators (Syrphidae and Lepidoptera). All surveys were carried once per field between 08:35 and 18:35 in the period 19th June to 2nd August in both years following focal fields’ sunflower bloom, and ensuring that weather standards established by Pollard & Yates [59] were fulfilled (temperature: 31.9 ± 1.9°C; wind: 3 ± 2.9 km/h). Visitation rates, expressed as number of pollinator visits per plant and hour, were derived from the total number of observed visits. The field studies did not involve endangered or protected species.

### Sunflower production

From each sunflower head, achenes were collected and separated into either ‘fully developed’ and ‘empty’ ones (containing only the embryo sac), and subsequently each group was weighted and counted using a photoelectronic seed counter (Pfeuffer Contador). Seed set was expressed as the percentage of filled achenes over the total number of achenes. Flour humidity and oil content of each head were measured from a freshly milled random sub-sample of 5 grams of filled achenes from each flower head using near-infrared transmittance technology (FOSS Infratec^™^ 1241 Grain Analyser).

Additionally, to determine whether pollination level affected the chemical composition of sunflower oil, only for 2014 fields, flour samples of each treatment-field combination were pooled and fatty acid methyl esters determined with a gas chromatograph. Nowadays, breeding programs have led to two main groups of oilseed sunflower hybrids depending on their oil quality: high-oleic and linoleic. In order to take this into account, all cultivars were classified into these two groups (hereafter coded as ‘High-oleic’ and ‘Linoleic’) based on the technical brochures provided by the seed companies.

### Statistical analyses

In order to calculate the effect of pollinator visitation rates on seed set and oil content, a two-step analysis was implemented. In the first step, the cross-pollination dependence (CPD), defined as the difference between ‘Pollinator-excluded’ and ‘Pollen supplemented’ plants of each cultivar, was determined through two generalized linear mixed models (GLMMs) with a beta error distribution, using seed set and oil content as response variables; pollination treatment, cultivar, year and the interaction between treatment and cultivar as fixed effects; and plots nested within fields as random effects. The predicted CPD for each cultivar was computed from the GLMM models by calculating the least square means (lsmeans) of ‘Pollen supplemented’ and ‘Pollinator-excluded’ plants within each cultivar. CPD estimates for each cultivar were then used to model the effect of visitation rates and the effect of landscape metrics on sunflower seed set and oil content. To verify that there was no spatial autocorrelation among focal fields in terms of cross-pollination dependence, a Mantel test was performed using the mantel.rtest function of the package ade4 [60] with 9999 random permutations taking into account two distance matrices: one containing the spatial distances between the focal fields, and one containing the distances between the computed CPD values of the cultivars present in those fields.

In the second step, the effect of visitation rates on seed set and oil content was tested by comparing ‘Pollinator-excluded’ and ‘Open pollination’ plants. Zero visits were associated to the ‘Pollinator-excluded’ plants. Effect of visitation rates was modelled through GLMMs with a beta error distribution with log-transformed visitation rates, CPD of each cultivar and year as fixed effects and plots nested within fields as random effects.

Then, in order to test whether chemical composition of sunflower oil is affected by pollination level, we performed two beta regression models, one for each of the main fatty acids of sunflower oil (oleic and linoleic), with hybrid type (‘High-oleic’ and ‘Linoleic’), pollination treatment (‘Pollinator-excluded’, ‘Open pollination’ and ‘Pollen supplemented’) and their interaction as explanatory variables.

The existence of a pollination deficit was tested by fitting two GLMMs with a beta error distribution with seed set and oil content of ‘Open pollination’ and ‘Pollen supplemented’ plants (pooled per distance) as response variables, pollination treatment and year as fixed effects, and field as random term.

To test which of the local and landscape variables affected seed set, a two-step selection process was applied based on data from 2014. A GLMM with a beta error distribution was performed using field as random term (to account for plots being nested within fields) and seed set increment as response variable. Seed set increments were calculated as the difference between the average actual seed set value of ‘Open pollination’ plants per plot and the baseline level due to within-head selfing of that specific cultivar (previously estimated based on ‘Pollinator-excluded’ plants). The predictor variables used in the GLMMs were: type of adjacent SNH, distance from the focal side, CPD, influence of apiaries in the surrounding landscape extracted from the heatmaps and landscape proportion of the following classes: cereals, grassy forage crops, legumes, other annual crops, sunflower, urban areas, woody areal SNH (WA), woody linear SNH (WL), herbaceous areal SNH (HA), herbaceous linear SNH (HL). Firstly, to identify the relevant landscape predictors, we performed an automated model selection (dredge function [61]) based on Akaike’s information criterion for small sample sizes (AICc), with adjacent SNH, distance and CPD of each cultivar coerced to be present in all models as covariates, and the proportions of each land use type and the beehives heatmap value as fixed factors. From all the possible combinations of landscape parameters, we excluded those with collinear predictors (Section G.A in S1 Appendix). Only those landscape variables with a relative importance greater than 0.2 in the subset of models with ΔAICc < 5 were taken into account (see Section G.B in S1 Appendix for details). Secondly, a standard model selection procedure was performed, including adjacent field border typology, the CPD of each cultivar and the previously selected landscape variables as fixed effects, and field as random term.

All model assumptions were checked adopting the graphical validation procedures recommended by Zuur et al. [62]. Model comparison was based on likelihood ratio test with *χ*^2^ distribution and multiple pairwise comparisons on least-squares means with Tukey’s adjustment of the P value. Statistical analyses were performed in R 3.2.5 [63] using the packages glmmADMB [64, 65], MuMIn [61], lsmeans [66] and ade4 [60].

## Results

One of the 2014 fields with adjacent linear grassy SNH was severely damaged before samples could be collected and thus discarded. Altogether, 25 sunflower fields were evaluated scattered over an area of 647 km^2^.

### Sunflower visitors

In 2014, 1861 insect visits were recorded during 11 h and 20 min of observations: 1820 were honey bee visits, 18 bumble bee visits, 21 other wild bee visits, and 2 visits from other pollinators. In 2015, 590 visits were registered in 2 h and 40 min of observations: 554 were honey bee visits, 31 bumble bees, 5 other wild bees and no visits from other pollinators (see Table F in S1 Appendix for wild bee species abundance). For both years, honey bees were the most abundant insect pollinator of sunflower, with 97.8% and 93.9% of the visits respectively.

### Effect of visitation rates

**Fig 2:**
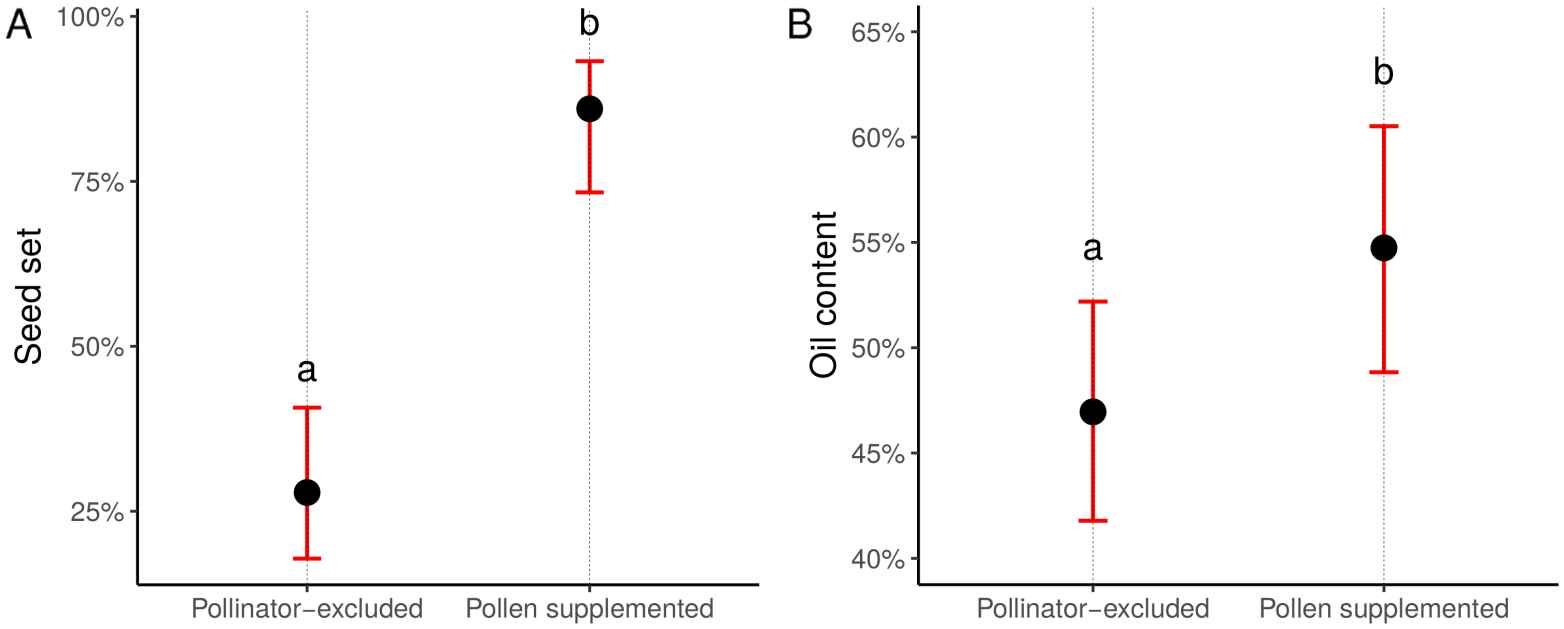
CPD of sunflower for (A) seed set, (B) oil content. Dots represent the predicted least-squares means of the GLMM averaged over the different cultivars. Bars represent the confidence intervals at the 95% level (back-transformed from the logit scale). Letters indicate statistically different groups at the 0.05 significance level. Tests were performed on the log odds ratio scale.

Since 20 plants were found damaged, in total, 189 ‘Pollinator-excluded’ heads and 191 ‘Pollen supplemented’ heads were included in the analysis to assess the CPD of cultivars. Seed set and oil content were strongly affected by pollination treatments: percentage of fully developed achenes and oil content of ‘Pollinator-excluded’ heads were significantly lower than those of ‘Pollen supplemented’ heads (seed set: z = -10.133, *p* < 0.001— Fig 2A; oil content: z = 5.483, *p* < 0.001— Fig 2B). This increase was consistent among all cultivars, although, in general, the self-fertile cultivars were less affected by the lack of cross-pollination than others (treatment—cultivar interaction: *χ*^2^ = 187.75, df = 12, *p* < 0.001 for seed set; *χ*^2^ = 120.75, df = 12, *p* < 0.001 for oil content; Table G in S1 Appendix). The CPD of each cultivar, computed from the GLMM models (Table 1), were then used in subsequent models.

**Table 1:** Least-squares means of seed set (%) and oil content (%) per cultivar. For each cultivar (row) the table reports the least square means of the two performed models: one testing the effect of pollination treatment (‘Pollinator-excluded’ vs. ‘Pollen supplemented’ plants) on seed set (columns 2-4; hereafter ‘CPD seed set’), and one on oil content (columns 5-7; hereafter ‘CPD oil content’). Standard errors are listed in parentheses.

	Seed set			Oil content		
Cultivar	Pollinator-excl.	Pollen suppl.	Δ cross-poll.	Pollinator-excl.	Pollen suppl.	Δ cross-poll.
Heliawin-KWS	47.7 (6.8)	86.3 (4.6)	38.6	48.9 (2.5)	53.1 (2.8)	4.2
Imeria-Caussade	47.0 (11.7)	83.7 (9.0)	36.8	52.0 (4.4)	56.6 (4.8)	4.6
Inostarck-Apsov	2.6 (1.5)	79.4 (11.0)	76.8	43.0 (4.3)	53.2 (5.0)	10.3
Inotop-Apsov	32.5 (10.4)	77.3 (11.2)	44.8	44.3 (4.3)	53.2 (4.9)	8.9
Klarika Cl-Caussade	29.6 (8.6)	88.9 (6.0)	59.2	47.4 (3.8)	53.6 (4.2)	6.2
LG 55.57 HO-LG	9.3 (3.8)	86.9 (7.0)	77.7	41.4 (3.9)	57.0 (4.4)	15.7
LG 56.56 HO-LG	8.4 (4.0)	86.3 (8.2)	78.0	41.3 (4.2)	54.5 (4.9)	13.3
Mas 83.R-Maisadour	17.2 (6.9)	83.2 (9.3)	66.1	47.5 (4.4)	56.4 (4.9)	8.9
Mas 86.OL-Maisadour	72.6 (9.6)	88.5 (7.0)	16.0	51.3 (4.4)	54.8 (4.9)	3.5
P64HE39-Pioneer	27.9 (8.1)	82.1 (8.2)	54.2	47.2 (3.7)	53.1 (4.2)	5.9
PR64H41-Pioneer	56.2 (10.7)	90.3 (5.6)	34.1	48.2 (4.0)	53.8 (4.4)	5.6
PR64H42-Pioneer	46.8 (10.8)	88.8 (6.3)	42.0	47.1 (4.0)	52.5 (4.5)	5.4
Sangria CS-Caussade	29.7 (9.1)	90.4 (5.6)	60.7	51.1 (4.0)	59.6 (4.3)	8.5

**Fig 3:**
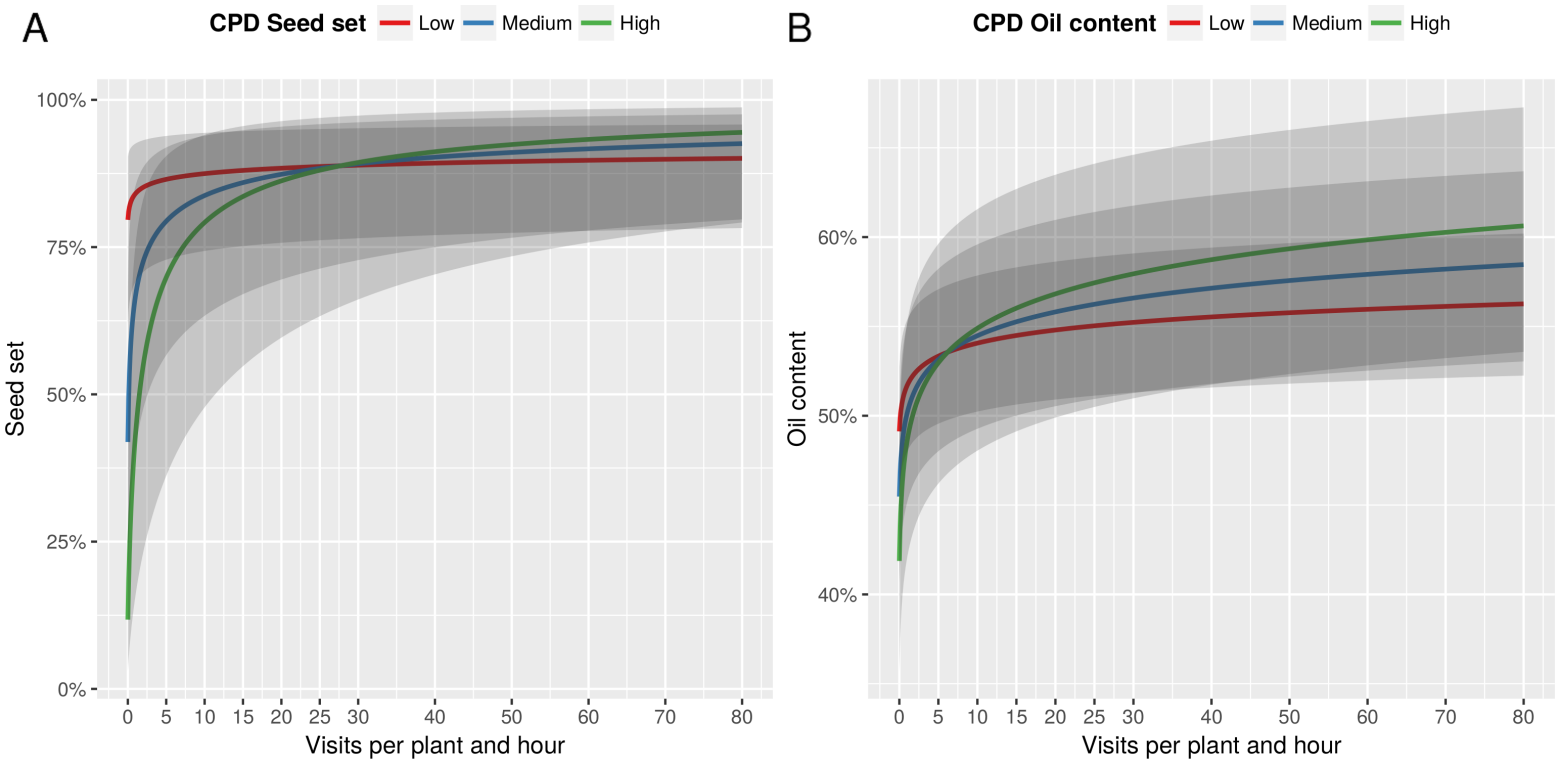
Effect of visitation rates and CPD on seed set and oil content. (A) Effect of visitation rates on seed set over three unique values of ‘CPD seed set’ (‘Low’: 15% CPD; ‘Medium’: 45% CPD; ‘High’: 75% CPD) chosen to represent this continuous variable in two dimensions; (B) Effect of visitation rates on seed set over three unique values of ‘CPD oil content’ (‘Low’: 5% CPD; ‘Medium’: 10% CPD; ‘High’: 15% CPD) chosen to represent this continuous variable in two dimensions. Results are based on GLMM. Solid lines show predicted values, grey ribbons are upper and lower confidence intervals at the 95% level.

For the visitation rate model, the visitation data previously presented were used. The low values of wild pollinators impeded a separate analysis for wild and managed pollinators nor could the existence of an interaction between the two groups be tested. In total, 792 sunflower heads were included in the analysis. Year of sampling was not significant for any of the response variables and thus removed from the final models (seed set model: *χ*^2^ = 2.372, df = 1, *p* = 0.12; oil content model: *χ*^2^ = 0.36, df = 1, *p* = 0.55). Insect visitation significantly increased seed set, but these gains differed from cultivar to cultivar depending on their CPD (interaction visitation rate-CPD Seed set: *χ*^2^ = 214.33, df = 1, *p* < 0.001; Table H in S1 Appendix). Cultivars with lower levels of self-fertility showed a greater response to insect visitation and suffered yield losses at low levels of visitation rates (Fig 3A). Analogously, higher visitation rates also resulted in increased oil content of filled achenes, with cultivars responding differently depending on their degree of CPD (interaction visitation rate-CPD Oil content: *χ*^2^ = 172.24, df = 1, *p* < 0.001; Table H in S1 Appendix). Cultivars with greater dependence showed greater vulnerability to lack of pollinators, but in case cross-pollination needs were fulfilled they produced higher oil quantities (Fig 3B).

Regarding the fatty acid composition of sunflower oil, high-oleic hybrids showed on average 90.4% and 2.9% of oleic and linoleic acids respectively, whereas linoleic hybrids displayed a 35.6% of oleic acid and a 55.1% of linoleic. The amount of both fatty acids was only dependent on the hybrid type (oleic model: *χ*^2^ = 134.73, df = 1, *p* < 0.001; linoleic model: *χ*^2^ = 107.22, df = 1, *p* < 0.001), and oil composition was not affected by cross-pollination through insect visits (oleic model: *χ*^2^ = 0.86, df = 2, *p* = 0.65; linoleic model: *χ*^2^ = 1.96, df = 2, *p* = 0.38) or the interaction pollination treatment-hybrid type (oleic model: *χ*^2^ = 3.12, df = 2, *p* = 0.21; linoleic model: *χ*^2^ = 0.32, df = 2, *p* = 0.85).

### Pollination deficit

**Fig 4:**
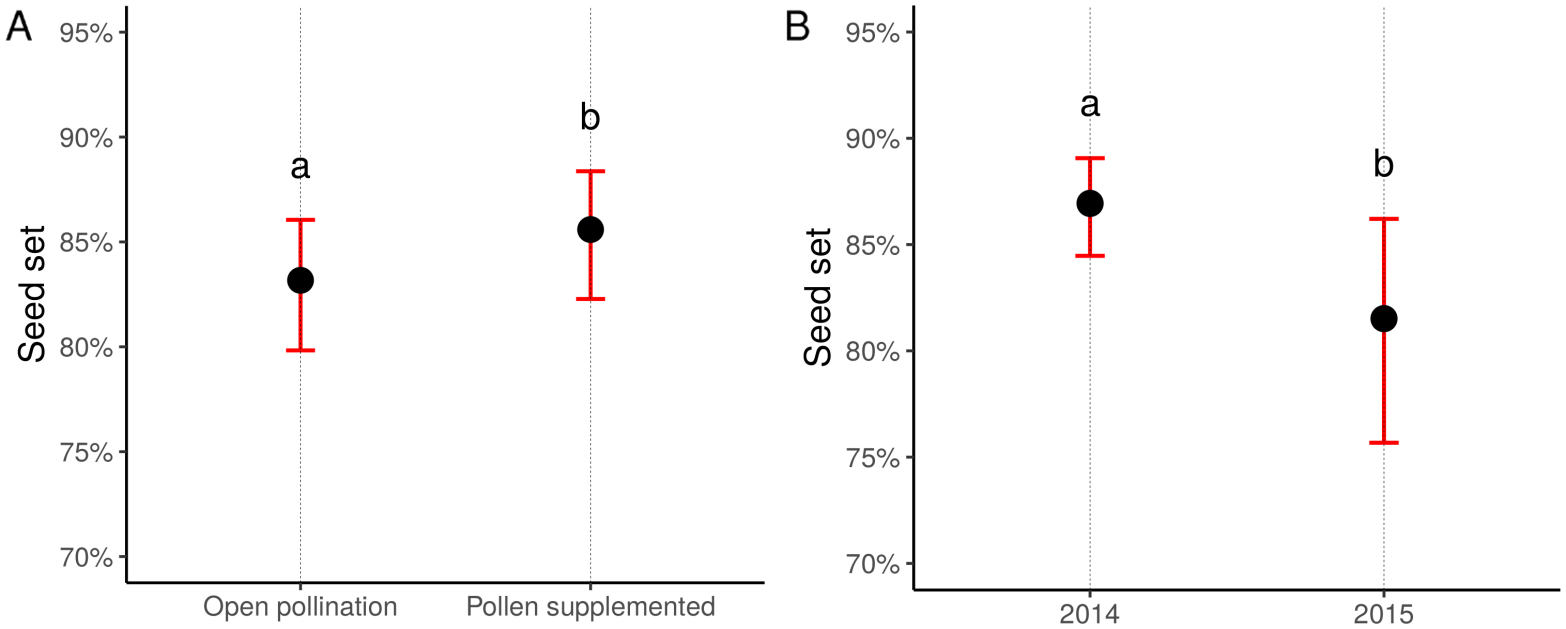
Effect on seed set (% of fully developed achenes) of (A) pollination treatment, (B) year. Dots represent the predicted least-squares means of the GLMM averaged over (A) years and (B) pollination treatments. Bars represent the confidence intervals at the 95% level (back-transformed from the logit scale). Letters indicate statistically different groups at the 0.05 significance level. Tests were performed on the log odds ratio scale.

The pollen supplementation experiment revealed that seed set was significantly affected by pollination treatment (*χ*^2^ = 11.886, df = 1, *p* = 0.0006) and year (*χ*^2^ = 5.074, df = 1, *p* = 0.0243). ‘Pollen supplemented’ plants showed on average 2.4% more filled achenes than ‘Open pollination’ ones (z = 3.521, *p* = 0.0004, Fig 4A). Seed set declined in 2015, and the percentage of fully developed achenes was on average 5.4% lower than in the previous year (z = -2.373, *p* = 0.0176, Fig 4B). Neither pollination treatment (*χ*^2^ = 1.356, df = 1, *p* = 0.24) nor year (*χ*^2^ = 0.122, df = 1, *p* = 0.72) affected the oil content of filled achenes.

### Landscape drivers of pollination service delivery

**Fig 5:**
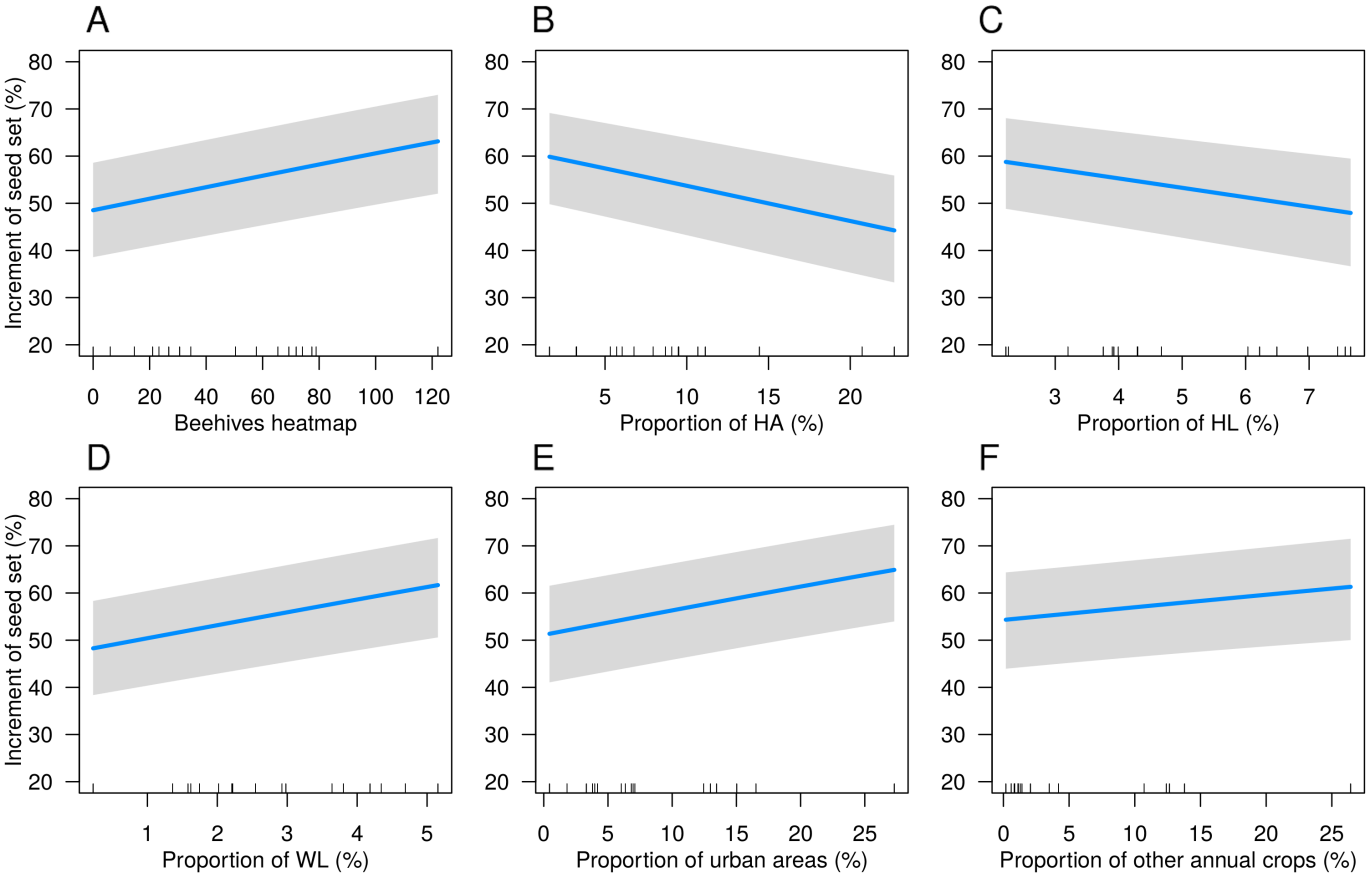
Effect of landscape variables on pollination success. Pollination success (expressed as mean increment of seed set) is modelled in relation to (A) beehives heatmap, (B) proportion of herbaceous areal SNHs, (C) proportion of herbaceous linear SNHs, (D) proportion of woody linear SNHs, (E) proportion of urban areas, (F) proportion of other annual crops. Results are based on GLMM. Solid lines show predicted values, grey ribbons are upper and lower confidence intervals at the 95% level. Plots are constructed holding all other variables constant in their median value for numeric variables and most common category for factors.

Pollination service delivery (measured as seed set increment) was not affected by the proportion of legume crops (*χ*^2^ = 2.214, df = 1, *p* = 0.14) nor by the distance to the border (*χ*^2^ = 2.322, df = 3, *p* = 0.51), and these variables were thus removed from the minimal adequate models. Pollination service delivery increased significantly with the beehives heatmap value (*χ*^2^ = 20.682, df = 1, *p* < 0.001) and with the proportion of WL elements (*χ*^2^ = 17.906, df = 1, *p* < 0.001), urban areas (*χ*^2^ = 17.826, df = 1, *p* < 0.001) and other annual crops (*χ*^2^ = 7.238, df = 1, *p* < 0.01). It was negatively affected by the proportion of HA (*χ*^2^ = 20.234, df = 1, *p* < 0.001) and HL (*χ*^2^ = 13.830, df = 1, *p* < 0.001). Seed set was also significantly affected by the type of adjacent SNH (*χ*^2^ = 16.296, df = 2, *p* < 0.001), with ‘woody SNH’ resulting in reduced seed set compared to ‘herbaceous SNH’ (z = 3.570, *p* < 0.01) and ‘no SNH/control’ (z = 4.939, *p* < 0.001) (Fig 5; Table L in S1 Appendix).

## Discussion

### Insect pollinators

In accordance with previous findings (carried out both in the same and in other areas) [30, 44, 45, 67, 68] our data confirm that in the study area honey bee is the most abundant pollinator of sunflower. Although in other contexts high numbers of other insects visiting sunflower have been observed [28, 36, 37], the disproportionate prevalence of honey bees recorded in this study can be explained by the historical [42, 43] and ongoing nomadism conducted by local bee-keepers to produce sunflower honey, which is a common practice in Central Italy. On the other hand, the very low visitation rates by wild bees, hoverflies and butterflies might have been caused by: (i) the observation plot method, which commonly underestimates bee species richness in agricultural habitats [69], but, in the case of the present work, may also have introduced a possible bias in bee abundance; (ii) a truly scarce presence of these groups in the study area; or (iii) a shifting due to competition with honey bees. The concern about these presumably under-sampled groups is based on findings from other studies which demonstrated that they may have a greater pollination efficiency or may improve the efficacy of pollination by honey bees [36]. However, the great prevalence of honey bees and the good fit of the data with the measured visitation rates, leads us to believe that the contribution of wild pollinators is rather limited in case honey bees are present. In spite of everything, since flower visitation may be a poor proxy for pollination effectiveness [70] and we did not measure pollinators’ visiting time, number of florets touched, or single visit pollen deposition, caution is needed in data interpretation.

### Effect of visitation rates

The obtained results demonstrate that cross-pollination improves seed set and oil content of sunflower, resulting in higher yields per ha. The degree of CPD varies among cultivars, suggesting that breeding programs have succeeded (at least partially) in raising the level of self-fertility. These findings are in line with previous studies, which state that cross-pollination exerts a direct [30, 68] or indirect (by mitigating reductions in seed set due to adverse abiotic conditions [34]) positive effect on sunflower productivity.

Our model indicates that visitation rates can enhance both seed set and oil content. Sunflower cultivars having lower levels of self-fertility benefit from increased seed set compared to self-fertile cultivars when they receive over ∼ 25 visits per head per hour, and produce higher oil content when they receive over ∼ 7.5 visits per head per hour. In fact, the level of seed set and oil content of varieties with a high level of self-fertility is never as high as properly pollinated varieties with a lower level of self-fertility (Fig 3). This trend poses the dilemma of either ensuring pollination service and taking advantage of it using dependent cultivars, or continuing to develop cultivars with a high self-pollinating ability at the price of losing a bit of the maximum potential productivity of sunflower. At least from an agronomic perspective, both strategies are reasonable and not exclusive.

Moreover, and contrary to our hypothesis, oil composition seems to be only genotype dependent and is not altered by the levels of cross-pollination. Thus, only the selection of a high oleic sunflower variety will drive the quality of the oil produced.

### Pollination deficit

In the Mediterranean cropping system under study, there was evidence of a pollination deficit which reduced crop productivity. On average, the seed set was 2.4% below its maximum. This pollination deficit may be considered marginal, but any short-term effect (e.g. bad weather, competitive bloom) that lowers visit densities may actually lead to a more severe pollen limitation. In order to ameliorate sunflower pollination in the area, two strategies could be pursued: (i) enhance the presence of managed honey bees in sunflower fields; and (ii) manage the surrounding landscape, and especially SNHs, in order to increase the visits of wild bees in sunflower fields. The first strategy further enhances the reliance on a single species (honey bee), and poses the risk of an insufficient pollination level if this species faces important declines. Therefore, the second strategy may be more rewarding, provided that future research is able to determine the efficacy of individual wild pollinators (field scale) and their overall crop pollination potential (landscape scale) in order to clarify whether they can increase sunflower pollination to its upper limit. In any case, the selection among the different strategies to maintain/ameliorate crop pollination needs to be carefully evaluated based on ecological, economical and agronomical considerations.

### Landscape drivers of pollination service delivery

From a landscape perspective, pollination service is the result of the interactions between land use patterns, local pollinator community and managed pollinators. Landscape patches may support pollinators by providing nesting opportunities, floral resources or shelter [22], but they may also compete with each other for pollinators if they have overlapping flowering periods. On the one hand, late mass-flowering crops that are to some extent self-incompatible (as sunflower) need to fulfil their cross-pollination requirements with high densities of pollinators, but these high numbers can only be sustained in the agroecosystem if there are enough early-flowering resources (e.g. oilseed rape or natural trees) at the beginning of the season. On the other hand, SNHs can provide suitable nesting sites and continuous floral resources preventing gaps in food supply in-between crop blooms thus increasing the abundance of pollinators, but that, as a drawback, may reduce pollination service delivery by diluting pollinator densities if they co-flower with the crop of interest. Sunflower and other mass-flowering crops generate huge pollination demands during short periods, forcing them to mainly rely on social pollinator species such as bumble bees and honey bees.

These bees, due to their high resource requirements, are especially sensitive to discontinuity of floral resources [67]. Several studies have shown that SNHs (like grasslands, hedgerows, fallows) enhance bumble bee densities [71, 72], whereas other studies stated that these were more related to early mass-flowering crops [73] or a combination of both SNHs and early mass-flowering crops [67]. Likewise, domestic gardens present in urban areas, especially if they are embedded in a matrix of intensively managed agricultural patches, may also positively affect abundance and richness of wild bees due to their high floral density and diversity together with greater nesting sites [71, 74]. In line with these studies, we found that the proportion of woody linear SNHs, other annual crops (e.g. oilseed rape) and urban areas enhanced the pollination service. In the study region, woody linear elements are mainly composed by early flowering species (e.g. *Prunus* spp, *Acer* spp, *Ulmus* spp, *Rubus* spp or *Crataegus* spp [75]), which may increase colony size of social bees by offering key floral resources during the beginning of colony development. On the contrary, our results also indicate that the presence of woody areal SNH does not affect sunflower pollination. This is in line with Hannon et al. [76] who reported that other habitats (such as agricultural areas and hedgerows) can be considered as better foraging areas than woodlots because they provide more accessible floral resources throughout the year.

At the same time, herbaceous SNHs exerted a negative influence on crop pollination. These elements are usually composed by a more diverse plant community (e.g. *Trifolium* spp, *Convolvulus arvensis*, *Dipsacus fullonum*, *Lythrum salicaria*, *Cichorium intybus*), increasing the probability of flowering periods that overlap with sunflower bloom, resulting in a dilution of crucial generalist pollinators [77–79]. Lastly, our findings confirm that the number of beehives present in the surroundings positively influences pollination of sunflower.

Finally, at the local scale, our analysis suggest that, as SNH-social bees dynamics work at a larger scales [80, 81], the presence of a SNH as nearby element might not result in any benefit, while, at least for hedgerows, may exert a negative influence on crop yield via direct competition for light and nutrients with sunflowers at the field border [82]. The overall effect of the ‘woody SNH’ on total yield of the adjacent field will depend on the field size and hedgerow height (higher competition for taller lines of trees). The larger the field, the smaller the relative hedgerow effect. Since the fields in the study region are relatively small, the edge effect of hedgerows visible in the first sampling point (2 m distance from the focal SNH) may have had a non-negligible effect on the overall yield.

## Conclusions and implications

This study confirms that sunflower yield is still greatly dependent on cross-pollination events, with even self-fertile cultivars showing a yield increase due to insect mediated cross-fertilisation. These events are exclusively driven by insect visitors (mainly honey bees). Therefore, in order to guarantee the productivity of currently used sunflower cultivars, it seems essential to ensure a pollinator community that is able to fulfil such cross-pollination needs. On the other hand, oil quality does not vary when cross-pollination events occur.

It also demonstrates that in Central Italy there is limited evidence of pollination deficits substantially reducing crop productivity. Nevertheless, the reliance on a single species (which is currently threatened by multiple stressors) could pose some risks for the future.

Finally, our findings suggest that the implementation of hedgerows within the agricultural matrix, aiming at supporting pollination services, deserves particular attention in agricultural policies. These elements may offer early flower resources to social bees, boosting their presence in subsequent sunflower fields, but as a drawback, they may directly compete for light and soil nutrients with the crop at the field edge, affecting yield especially in small fields (as it is the case in the study area).

In view of the results, some relatively simple and complementary measures may be taken to prevent future problems: (i) support healthy honey bee colonies and local communities of wild bees throughout diversified and balanced landscapes containing various SNH typologies and crop types with early and late flowering species, (ii) implement incentive schemes to support the economic activity of bee-keepers to ensure a homogeneous distribution of honey bee colonies on the territory.

## Supporting information

S1 Appendix Supporting appendix. Supplementary information associated with this article can be found in S1 Appendix.
(PDF)

## References

[1] Garibaldi LA, Aizen MA, Klein AM, Cunningham SA, Harder LD. Global growth and stability of agricultural yield decrease with pollinator dependence. Proceedings of the National Academy of Sciences of the United States of America. 2011;108(14):5909–5914. 10.1073/pnas.1012431108 21422295 PMC3078347

[2] Ellis JD, Evans JD, Pettis J. Colony losses, managed colony population decline, and Colony Collapse Disorder in the United States. Journal of Apicultural Research. 2010;49(1):134–136. 10.3896/IBRA.1.49.1.30

[3] Potts SG, Roberts SPM, Dean R, Marris G, Brown MA, Jones R, et al Declines of managed honey bees and beekeepers in Europe. Journal of Apicultural Research. 2010;49(1):15–22. 10.3896/IBRA.1.49.1.02

[4] Goulson D, Lye GC, Darvill B. Decline and Conservation of Bumble Bees. Annual Review of Entomology. 2008;53(1):191–208. 10.1146/annurev.ento.53.103106.093454 17803456

[5] Biesmeijer JC, Roberts SPM, Reemer M, Ohlemüller R, Edwards M, Peeters T, et al Parallel declines in pollinators and insect-pollinated plants in Britain and the Netherlands. Science (New York, NY). 2006;313(5785):351–4. 10.1126/science.1127863 16857940

[6] Potts SG, Biesmeijer JC, Kremen C, Neumann P, Schweiger O, Kunin WE. Global pollinator declines: Trends, impacts and drivers. Trends in Ecology and Evolution. 2010;25(6):345–353. 10.1016/j.tree.2010.01.007 20188434

[7] Koh I, Lonsdorf EV, Williams NM, Brittain C, Isaacs R, Gibbs J, et al Modeling the status, trends, and impacts of wild bee abundance in the United States. Proceedings of the National Academy of Sciences. 2016;113(1):140–145. 10.1073/pnas.1517685113 PMC4711882 26699460

[8] Goulson D, Nicholls E, Botías C, Rotheray EL. Bee declines driven by combined stress from parasites, pesticides, and lack of flowers. Science. 2015;347(6229):1–16. 10.1126/science.1255957 25721506

[9] Vaissière B, Freitas BM, Gemmill-Herren B. Protocol to detect and assess pollination deficits in crops: a handbook for its use. FAO; 2011 Available from: http://www.fao.org/docrep/013/i1929e/i1929e00.htm.

[10] Aizen MA, Harder LD. The Global Stock of Domesticated Honey Bees Is Growing Slower Than Agricultural Demand for Pollination. Current Biology. 2009;19(11):915–918. 10.1016/j.cub.2009.03.071 19427214

[11] National Research Council. Status of Pollinators in North America. Washington, D.C.: National Academies Press; 2007 Available from: http://www.nap.edu/catalog/11761.

[12] Steffan-Dewenter I, Potts SG, Packer L. Pollinator diversity and crop pollination services are at risk. Trends in ecology & evolution. 2005;20(12):651–652. 10.1016/j.tree.2005.09.004 16701452

[13] Aizen MA, Garibaldi LA, Cunningham SA, Klein AM. Long-term global trends in crop yield and production reveal no current pollination shortage but increasing pollinator dependency. Current biology. 2008;18(20):1572–5. 10.1016/j.cub.2008.08.066 18926704

[14] Ghazoul J. Buzziness as usual? Questioning the global pollination crisis. Trends in Ecology and Evolution. 2005;20(7):367–373. 10.1016/j.tree.2005.04.026 16701398

[15] Breeze TD, Vaissière BE, Bommarco R, Petanidou T, Seraphides N, Kozák L, et al Agricultural Policies Exacerbate Honeybee Pollination Service Supply-Demand Mismatches Across Europe. PLoS ONE. 2014;9(1):1–8. 10.1371/journal.pone.0082996 PMC3885438 24421873

[16] MacIvor JS. Cavity-nest boxes for solitary bees: a century of design and research. Apidologie. 2017;48(3):311–327. 10.1007/s13592-016-0477-z

[17] Kennedy CM, Lonsdorf E, Neel MC, Williams NM, Ricketts TH, Winfree R, et al A global quantitative synthesis of local and landscape effects on wild bee pollinators in agroecosystems. Ecology Letters. 2013;16(5):584–599. 10.1111/ele.12082 23489285

[18] Garibaldi LA, Steffan-Dewenter I, Kremen C, Morales JM, Bommarco R, Cunningham SA, et al Stability of pollination services decreases with isolation from natural areas despite honey bee visits. Ecology Letters. 2011;14(10):1062–1072. 10.1111/j.1461-0248.2011.01669.x 21806746

[19] Scheper J, Holzschuh A, Kuussaari M, Potts SG, Rundlöf M, Smith HG, et al Environmental factors driving the effectiveness of European agri-environmental measures in mitigating pollinator loss—a meta-analysis. Ecology letters. 2013;16(7):912–920. 10.1111/ele.12128 23714393

[20] Tscharntke T, Clough Y, Wanger TC, Jackson L, Motzke I, Perfecto I, et al Global food security, biodiversity conservation and the future of agricultural intensification. Biological conservation. 2012;151(1):53–59. 10.1016/j.biocon.2012.01.068

[21] Park MG, Blitzer EJ, Gibbs J, Losey JE, Danforth BN. Negative effects of pesticides on wild bee communities can be buffered by landscape context. Proceedings of the Royal Society of London B: Biological Sciences. 2015;282(1809):20150299 10.1098/rspb.2015.0299 PMC4590442 26041355

[22] Morrison J, Izquierdo J, Plaza EH, González-Andújar JL. The role of field margins in supporting wild bees in Mediterranean cereal agroecosystems: Which biotic and abiotic factors are important? Agriculture, Ecosystems and Environment. 2017;247(July):216–224. 10.1016/j.agee.2017.06.047

[23] Sarthou JP, Badoz A, Vaissière B, Chevallier A, Rusch A. Local more than landscape parameters structure natural enemy communities during their overwintering in semi-natural habitats. Agriculture, ecosystems & environment. 2014;194:17–28. 10.1016/j.agee.2014.04.018

[24] Holland JM, Douma JC, Crowley L, James L, Kor L, Stevenson DRW, et al Semi-natural habitats support biological control, pollination and soil conservation in Europe. A review. Agronomy for Sustainable Development. 2017;37(4):31 10.1007/s13593-017-0434-x

[25] Schirmel J, Albrecht M, Bauer PM, Sutter L, Pfister SC, Entling MH. Landscape complexity promotes hoverflies across different types of semi-natural habitats in farmland. Journal of Applied Ecology. 2018;55(4):1747–1758. 10.1111/1365-2664.13095

[26] Food and Agricultural Organization of the United Nations. FAOSTAT Statistical Databases; 2016. Available from: http://faostat.fao.org/.

[27] Cockerell TDA. Bees visiting Helianthus. The Canadian Entomologist. 1914;46(12):409–415. 10.4039/Ent46409-12

[28] Parker FD. Sunflower pollination: abundance, diversity and seasonality of bees and their effect on seed yields. Journal of Apicultural Research. 1981;20(1):49–61. 10.1080/00218839.1981.11100473

[29] Degrandi-hoffman G, Watkins JC. The foraging activity of honey bees Apis mellifera and non- Apis bees on hybrid sunflowers (Helianthus annuus) and its influence on cross-pollination and seed set. Journal of Apicultural Research. 2000;39(1-2):37–45. 10.1080/00218839.2000.11101019

[30] Carvalheiro LG, Veldtman R, Shenkute AG, Tesfay GB, Pirk CWW, Donaldson JS, et al Natural and within-farmland biodiversity enhances crop productivity. Ecology Letters. 2011;14(3):251–259. 10.1111/j.1461-0248.2010.01579.x 21244594

[31] Sardiñas HS, Kremen C. Pollination services from field-scale agricultural diversification may be context-dependent. Agriculture, Ecosystems & Environment. 2015;207(0):17–25.

[32] Sáez A, Sabatino M, Aizen MA. Interactive effects of large- and small-scale sources of feral honey-bees for sunflower in the Argentine Pampas. PLoS ONE. 2012;7(1):e30968 10.1371/journal.pone.0030968 22303477 PMC3267740

[33] Macchia M, Vicentini G. Caratteristiche degli acheni di girasole (*Helianthus annuus* L.) localizzati in settori diversi della calatide. L’agricoltura italiana. 1974;74:116–122.

[34] Degrandi-Hoffman G, Chambers M. Effects of Honey Bee (Hymenoptera: Apidae) Foraging on Seed Set in Self-fertile Sunflowers (*Helianthus annuus* L). Environmental entomology. 2006;35(4):1103–1108. 10.1603/0046-225X-35.4.1103

[35] Susic Martin C, Farina WM. Honeybee floral constancy and pollination efficiency in sunflower (*Helianthus annuus*) crops for hybrid seed production. Apidologie. 2015; p. 161–170.

[36] Greenleaf SS, Kremen C. Wild bees enhance honey bees’ pollination of hybrid sunflower. Proceedings of the National Academy of Sciences of the United States of America. 2006;103(37):13890–13895. 10.1073/pnas.0600929103 16940358 PMC1564230

[37] Mallinger R, Prasifka J. Benefits of insect pollination to confection sunflowers differ across plant genotypes. Crop Science. 2017;57(6):3264–3272. 10.2135/cropsci2017.03.0148

[38] Hurd PD Jr, LeBerge WE, Linsley EG. Principal sunflower bees of North America with emphasis on the southwestern United States (Hymenoptera, Apoidea). Smithsonian contributions to zoology. 1980;310:1–158. 10.5479/si.00810282.310

[39] Minckley RL, Wcislo WT, Yanega D, Buchmann SL. Behavior and Phenology of a Specialist Bee (Dieunomia) and Sunflower (Helianthus) Pollen Availability. Ecology. 1994;75(5):1406–1419. 10.2307/1937464

[40] Nderitu J, Nyamasyo G, Kasina M, Oronje ML. Diversity of sunflower pollinators and their effect on seed yield in Makueni District, Eastern Kenya. Spanish Journal of Agricultural Research. 2008;6(2):271–278. 10.5424/sjar/2008062-318

[41] Pisanty G, Klein AM, Mandelik Y. Do wild bees complement honeybee pollination of confection sunflowers in Israel? Apidologie. 2014;45(November 2015):235–247. 10.1007/s13592-013-0242-5

[42] Burmistrov AN. La valeur mellifère de quelques espèces de l’Helianthe. In: XX Apicultural International Congress Apimondia. Bucharest; 1965. p. 342–345.

[43] Ricciardelli D’albore G. L’importanza delle colture di *Helianthus annuus* L. per la produzione di miele e polline. L’apicoltore moderno. 1976;(67):109–115.

[44] Frediani D. Il ruolo delle api nell’impolinazione del girasole (*Heliantus annuus* L.) in Italia centrale. In: Simposio internazione di apicoltura. Torino; 1972.

[45] Ricciardelli d’Albore G. Osservazioni sui pronubi del girasole (*Helianthus annuus* L.) in Umbria. Redia giornale di zoologia. 1982;.

[46] Frediani D, Pinzauti M. Influenza dell’impollinazione entomofila sulla produzione dei semi nel girasole. L’apicoltore moderno. 1978;69(4):109–113.

[47] Quinet M, Jacquemart AL. Cultivar placement affects pollination efficiency and fruit production in European pear (*Pyrus communis*) orchards. European Journal of Agronomy. 2017;91(September):84–92. 10.1016/j.eja.2017.09.015

[48] Klatt BK, Holzschuh A, Westphal C, Clough Y, Smit I, Pawelzik E, et al Bee pollination improves crop quality, shelf life and commercial value. Proceedings of the Royal Society of London B: Biological Sciences. 2014;281(1775):20132440 10.1098/rspb.2013.2440 PMC3866401 24307669

[49] Bommarco R, Marini L, Vaissière BE. Insect pollination enhances seed yield, quality, and market value in oilseed rape. Oecologia. 2012;169(4):1025–1032. 10.1007/s00442-012-2271-6 22311256

[50] Alberio C, Izquierdo NG, Galella T, Zuil S, Reid R, Zambelli A, et al A new sunflower high oleic mutation confers stable oil grain fatty acid composition across environments. European Journal of Agronomy. 2016;73:25–33. 10.1016/j.eja.2015.10.003

[51] Miller JF, Zimmerman DC, Vick BA. Genetic Control of High Oleic Acid Content in Sunflower Oil. Crop Science. 1987;27:923–926. 10.2135/cropsci1987.0011183X002700050019x

[52] Marrero HJ, Medan D, Zarlavsky GE, Torretta JP. Agricultural land management negatively affects pollination service in Pampean agro-ecosystems. Agriculture, Ecosystems and Environment. 2016;218:28–32. 10.1016/j.agee.2015.10.024

[53] Cong RG, Smith HG, Olsson O, Brady M. Managing ecosystem services for agriculture: Will landscape-scale management pay? Ecological Economics. 2014;99:53–62. 10.1016/j.ecolecon.2014.01.007

[54] Ricketts TH, Regetz J, Steffan-Dewenter I, Cunningham SA, Kremen C, Bogdanski A, et al Landscape effects on crop pollination services: are there general patterns? Ecology Letters. 2008;11(5):499–515. 10.1111/j.1461-0248.2008.01157.x 18294214

[55] Jeanneret P, Albrecht M, Entling M, Giffard B, Heijne B, Helsen H, et al. Report on methods to assess ecosystem services. EU FP7 QUESSA project Deliverable D3.1; 2013. Available from: http://docs.wixstatic.com/ugd/3ccd83_89260c6807e8477e9dd631e590c84d7b.pdf.

[56] Regione Toscana—Sistema Informativo Territoriale ed Ambientale. GEOscopio WMS; 2016. Available from: http://www.regione.toscana.it/-/geoscopio-wms.

[57] QGIS Development Team. QGIS Geographic Information System; 2016. Available from: http://qgis.osgeo.org.

[58] Greenleaf SS, Williams NM, Winfree R, Kremen C. Bee foraging ranges and their relationship to body size. Oecologia. 2007;153(3):589–596. 10.1007/s00442-007-0752-9 17483965

[59] Pollard E, Yates TJ. Monitoring butterflies for ecology and conservation: the British Butterfly Monitoring Scheme. Springer Netherlands; 1993.

[60] Dray S, Dufour AB. The ade4 package: implementing the duality diagram for ecologists. Journal of Statistical Software. 2007;22(4):1–20. doi: 10.18637/jss.v022.i04

[61] Barton K. MuMIn: Multi-Model Inference; 2016. Available from: https://cran.r-project.org/package=MuMIn.

[62] Zuur AF, Ieno EN, Walker N, Saveliev AA, Smith GM. Mixed effects models and extensions in ecology with R Statistics for Biology and Health. Springer-Verlag New York; 2009 Available from: http://link.springer.com/10.1007/978-0-387-87458-6.

[63] R Core Team. R: A Language and Environment for Statistical Computing; 2016. Available from: https://www.r-project.org/.

[64] Fournier DA, Skaug HJ, Ancheta J, Ianelli J, Magnusson A, Maunder MN, et al {AD Model Builder}: using automatic differentiation for statistical inference of highly parameterized complex nonlinear models. Optim Methods Softw. 2012;27:233–249. 10.1080/10556788.2011.597854

[65] Skaug H, Fournier D, Bolker B, Magnusson A, Nielsen A. Generalized Linear Mixed Models using ‘AD Model Builder’; 2016.

[66] Lenth RV. Least-Squares Means: The {R} Package {lsmeans}. Journal of Statistical Software. 2016;69(1):1–33. doi: 10.18637/jss.v069.i01

[67] Riedinger V, Renner M, Rundlöf M, Steffan-Dewenter I, Holzschuh A. Early mass-flowering crops mitigate pollinator dilution in late-flowering crops. Landscape Ecology. 2014;29(3):425–435. 10.1007/s10980-013-9973-y

[68] McGregor SE. Insect pollination of cultivated crop plants. vol. 496 Agricultural Research Service, US Department of Agriculture; 1976.

[69] Westphal C, Bommarco R, Carré G, Lamborn E, Morison N, Petanidou T, et al Measuring bee diversity in different European habitats and biogeographical regions. Ecological monographs. 2008;78(4):653–671. 10.1890/07-1292.1

[70] King C, Ballantyne G, Willmer PG. Why flower visitation is a poor proxy for pollination: Measuring single-visit pollen deposition, with implications for pollination networks and conservation. Methods in Ecology and Evolution. 2013;4(9):811–818. 10.1111/2041-210X.12074

[71] Ekroos J, Rundlöf M, Smith HG. Trait-dependent responses of flower-visiting insects to distance to semi-natural grasslands and landscape heterogeneity. Landscape Ecology. 2013;28(7):1283–1292. 10.1007/s10980-013-9864-2

[72] Rundlöf M, Nilsson H, Smith HG. Interacting effects of farming practice and landscape context on bumble bees. Biological Conservation. 2008;141(2):417–426. 10.1016/j.biocon.2007.10.011

[73] Westphal C, Steffan-Dewenter I, Tscharntke T. Mass flowering crops enhance pollinator densities at a landscape scale. Ecology Letters. 2003;6(11):961–965. 10.1046/j.1461-0248.2003.00523.x

[74] Samnegård U, Persson AS, Smith HG. Gardens benefit bees and enhance pollination in intensively managed farmland. Biological Conservation. 2011;144(11):2602–2606. 10.1016/j.biocon.2011.07.008

[75] Bartual AM. The role of vegetation composition and structure of seminatural habitat in determining their ecosystem service provisioning potential and the relation to pollination in sunflower [Ph.D. dissertation]. Scuola Superiore Sant’Anna. Pisa, Italy; 2016.

[76] Hannon LE, Sisk TD. Hedgerows in an agri-natural landscape: Potential habitat value for native bees. Biological Conservation. 2009;142(10):2140–2154. 10.1016/j.biocon.2009.04.014

[77] Delaplane KS, Mayer DR, Mayer DF. Crop pollination by bees. Cabi; 2000 Available from: https://www.cabi.org/cabebooks/ebook/20000709824.

[78] Di Pasquale G, Salignon M, Le Conte Y, Belzunces LP, Decourtye A, Kretzschmar A, et al Influence of Pollen Nutrition on Honey Bee Health: Do Pollen Quality and Diversity Matter? PLOS ONE. 2013;8(8). 10.1371/journal.pone.0072016 PMC3733843 23940803

[79] Tasei JN, Aupinel P. Nutritive value of 15 single pollens and pollen mixes tested on larvae produced by bumblebee workers (Bombus terrestris, Hymenoptera: Apidae). Apidologie. 2008;39(4):397–409. 10.1051/apido:2008017

[80] Westphal C, Steffan-Dewenter I, Tscharntke T. Bumblebees experience landscapes at different spatial scales: Possible implications for coexistence. Oecologia. 2006;149(2):289–300. 10.1007/s00442-006-0448-6 16758219

[81] Steffan-Dewenter I, Kuhn A. Honeybee foraging in differentially structured landscapes. Proceedings of the Royal Society of London B: Biological Sciences. 2003;270(1515):569–575. 10.1098/rspb.2002.2292 PMC1691282 12769455

[82] De Costa WAJM, Surenthran P. Tree-crop interactions in hedgerow intercropping with different tree species and tea in Sri Lanka: 1. Production and resource competition. Agroforestry Systems. 2005;63(3):199–209.

