# Supplementary material for: Local and landscape factors affect sunflower pollination in a Mediterranean agroecosystem
Source: PLoS One. 2018 Sep 27;13(9):e0203990. doi: 10.1371/journal.pone.0203990 (PMC6159865; doi:10.1371/journal.pone.0203990)
Supplement: S1 Appendix — Supplementary information associated with this article can be found in S1 Appendix. (PDF) [file pone.0203990.s001.pdf]

S1 Appendix

Supplementary information associated with "Local and landscape factors affect sunflower pollination in a Mediterranean agroecosystem"

Agustín M. Bartual[1*], Gionata Bocci[1], Simone Marini[1*], Anna Camilla Moonen[1]

1 Institute of Life Sciences, Scuola Superiore Sant'Anna, Pisa, Italy

* am.bartual@gmail.com (AMB); simone.marini@santannapisa.it (SM)

A Study sites

Study sites (figure A) were allocated along a gradient of landscape complexity ranging from very simple landscapes, mainly dominated by agricultural land with a very low proportion of SNHs, to very complex ones, mostly dominated by SNHs (table E). Simple and complex landscape sectors were selected in such a way that they were spatially interspersed throughout the region. Additionally, to avoid confounding effects between the local variable (adjoining SNH type) and landscape complexity, each category of adjoining SNH was spread along the gradient of SNH cover in the landscape as described in the core text. Details about focal field management are reported in Tables A, B and C.

(a) (b)

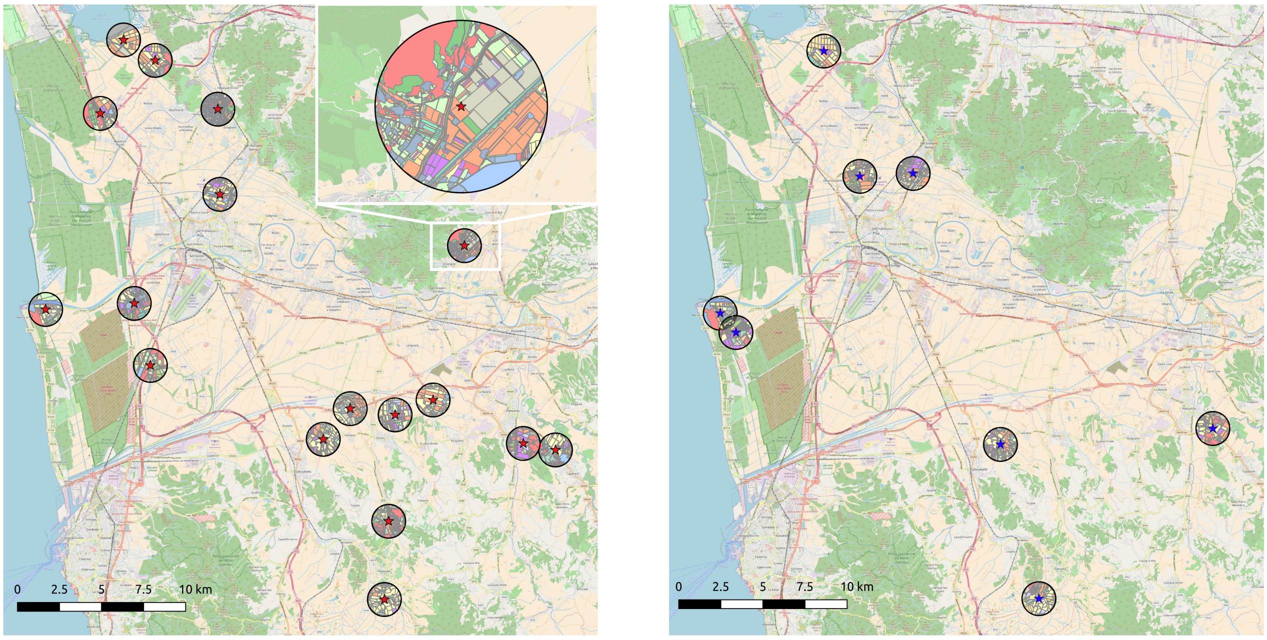

Fig A: Selected study sites in the Pisa plain, years 2014 and 2015: (a) The 17 (originally 18, see main document for further details) selected sunflower fields in 2014 (red stars) and the surrounding landscape in 1 km radius. In the upper right corner one landscape sector detail is provided: colours represent the different land uses present according to Table D (i.e. red: 'Woody areal SNH', green: 'Herbaceous areal SNH', orange: 'Sunflower', yellow: 'Cereals', blue: 'Urban areas'). (b) The eight selected sunflower fields in 2015 (blue stars) and the surrounding landscape sector in 1 km radius. Map in background is provided by ©OpenStreetMap contributors under CC BY-SA license.

Table A: Characteristics and broad agronomical information.

Focal field ID	Adjacent SNH	Size (ha)	Elevation (m a.s.l.)	Management type	Type of tillage
2014_1	CO	5.30	2.00	conventional	minimum tillage
2014_3	CO	3.10	5.50	conventional	minimum tillage
2014_4	HL	14.50	9.50	conventional	minimum tillage
2014_5	WL	2.90	55.00	conventional	inversion
2014_6	WL	4.10	3.00	conventional	inversion
2014_7	WL	13.40	0.70	conventional	minimum tillage
2014_8	HL	3.50	3.30	conventional	inversion
2014_9	HL	7.50	7.00	conventional	minimum tillage
2014_10	CO	5.30	12.50	conventional	inversion
2014_11	CO	2.20	33.00	organic	inversion
2014_12	WL	3.00	8.70	conventional	inversion
2014_13	WL	1.70	1.50	conventional	minimum tillage
2014_14	CO	11.30	0.00	conventional	minimum tillage
2014_15	WL	8.30	34.00	conventional	inversion
2014_16	HL	9.10	25.00	organic	inversion
2014_17	HL	7.00	0.50	conventional	minimum tillage
2014_18	CO	2.40	9.50	conventional	minimum tillage
2015_1	CO	13.20	2.10	conventional	inversion
2015_2	HL	21.70	2.00	conventional	inversion
2015_3	WL	15.50	0.60	conventional	inversion
2015_5	HL	2.20	0.80	conventional	minimum tillage
2015_6	HL	4.50	8.00	conventional	minimum tillage
2015_7	CO	6.60	0.70	conventional	minimum tillage
2015_8	HL	7.50	27.20	organic	inversion
2015_9	HL	2.50	48.50	conventional	inversion

Table B: Detailed agronomical information. Yield was calculated using plant density and average mass of achenes per plant; seed rate as declared by farmers; plant density was estimated by researchers using 3m X row distance frames (figure B); capitula lifespan is the number of days between opening of the first florets (R.5.1) to complete flowering and ray flowers wilting (R.6) [1].

Focal field ID	Crop variety cultivar	Sowing date	Harvest date	Yield (t/ha)	Seed rate (seeds/ha)	Density (plants/ha)	Capitula lifespan (d)	Flowering date
2014_1	P64HE39-Pioneer	05/05/2014	08/09/2014	4.60	60000	41667	10	07/07/2014
2014_3	LG 56.56 HO-LG	10/04/2014	03/09/2014	2.70	72000	51667	10	22/06/2014
2014_4	PR64H42-Pioneer	09/04/2014	09/09/2014	1.20	70000	27778	10	08/07/2014
2014_5	LG 55.57 HO-LG	10/05/2014	15/09/2014	4.50	75000	77222	10	15/07/2014
2014_6	Klarika Cl-Caussade	17/05/2014	15/09/2014	0.50	75000	34444	10	29/07/2014
2014_7	PR64H42-Pioneer	07/04/2014	08/09/2014	4.60	60000	62222	10	22/06/2014
2014_8	P64HE39-Pioneer	14/04/2014	05/09/2014	3.00	70000	65833	10	23/06/2014
2014_9	PR64H41-Pioneer	14/04/2014	03/09/2014	2.70	80000	53333	10	24/06/2014
2014_10	Acteon-KWS	09/05/2014	15/09/2014	4.50	50000	41667	10	08/07/2014
2014_11	Sangria CS-Caussade	06/05/2014	15/09/2014	2.00	70000	55000	10	17/07/2014
2014_12	P64HE39-Pioneer	05/05/2014	10/09/2014	4.00	75000	56111	10	07/07/2014
2014_13	Klarika Cl-Caussade	10/04/2014	27/09/2014	3.50	80000	55833	10	29/06/2014
2014_14	Imeria-Caussade	05/04/2014	02/09/2014	2.30	75000	77500	8	21/06/2014
2014_15	LG 55.57 HO-LG	15/04/2014	03/09/2014	4.00	72000	63333	10	24/06/2014
2014_16	Sangria CS-Caussade	24/04/2014	15/09/2014	2.00	70000	37778	10	08/07/2014
2014_17	PR64H41-Pioneer	03/04/2014	28/08/2014	3.20	65000	64444	10	15/06/2014
2014_18	Mas 83.R-Maisadour	24/05/2014	15/10/2014	2.00	70000	30556	10	27/07/2014
2015_1	P64HE39-Pioneer	20/03/2015	05/09/2015	2.30	70000	42778	8	20/06/2015
2015_2	P64HE39-Pioneer	20/04/2015	10/09/2015	3.00	75000	66667	8	01/07/2015
2015_3	P64HE39-Pioneer	20/05/2015	10/09/2015	3.00	75000	54444	8	29/06/2015
2015_5	Inostarck-Apsov	16/04/2015	22/09/2015	3.00	70000	34167	10	25/06/2015
2015_6	Klarika Cl-Caussade	08/05/2015	25/09/2015	2.90	75000	44444	6	02/07/2015
2015_7	Klarika Cl-Caussade	01/04/2015	01/09/2015	2.50	75000	76667	10	27/06/2015
2015_8	Inotop-Apsov	15/05/2015	10/09/2015	2.50	70000	34444	8	04/07/2015
2015_9	Mas 86.OL-Maisadour	05/04/2015	20/08/2015	2.90	70000	60556	8	04/07/2015

Table C: Management details. Total N fertilizer stands for total amount of nitrogenum; for insecticides, herbicides and molluscicides it is listed the active compound sprayed, the amount in kg/ha and the date of application. All information was provided by farmers.

Focal field ID	Tot N fertilizer (kg/ha)	Type of fertilizer	Insecticide	Herbicide 1	Herbicide 2	Weeds mechanical removal date	Molluscicide
2014_1	98.00	synthetic		tribenuron-methyl 0.025 kg/ha 05/06/2014		20/06/2014	
2014_3	100.00	synthetic	lambda-cyhalothrin 0.06 kg/ha 10/04/2014	pendimethalin 0.6205 kg/ha 14/04/2014	aclonifen 0.42 kg/ha 14/04/2014	30/05/2014	
2014_4	104.00	synthetic		tribenuron-methyl 0.03 kg/ha 01/06/2014		15/06/2014	
2014_5	84.00	synthetic				10/06/2014	
2014_6	92.00	synthetic		aclonifen 1.8 kg/ha 18/05/2014		30/06/2014	metaldehyde 0.05 kg/ha 25/05/2014
2014_7	98.00	synthetic		tribenuron-methyl 0.025 kg/ha 07/05/2014		30/05/2014	
2014_8	124.00	synthetic					
2014_9	118.00	synthetic		aclonifen 0.6 kg/ha 14/05/2014	quizalofop-p 0.05 kg/ha 01/06/2014	09/06/2014	metaldehyde 0.3 kg/ha 14/04/2014
2014_10	100.00	synthetic				15/05/2014	
2014_11	0.00	none				01/06/2014	
2014_12	71.40	synthetic		quizalofop-p 0.05 kg/ha 05/06/2014	tribenuron-methyl 0.025 kg/ha 05/06/2014		
2014_13	100.00	synthetic	tefluthrin 0.04 kg/ha 10/04/2014	s-metolachlor 0.96 kg/ha 10/05/2014	oxyfluorfen 0.24 kg/ha 10/05/2014		metaldehyde 0.05 kg/ha 10/04/2014
2014_14	58.00	synthetic					metaldehyde 0.15 kg/ha 08/04/2014
2014_15	100.00	synthetic	lambda-cyhalothrin 0.06 kg/ha 15/04/2014	pendimethalin 0.6205 kg/ha 20/04/2014	aclonifen 0.42 kg/ha 20/04/2014	30/05/2014	
2014_16	0.00	none				01/06/2014	
2014_17	98.00	synthetic		aclonifen 0.9 kg/ha 04/05/2014	quizalofop-p 0.05 kg/ha 08/05/2014	20/05/2014	metaldehyde 0.05 kg/ha 11/04/2014
2014_18	0.00	none					
2015_1	92.00	synthetic		aclonifen 1.2 kg/ha 21/03/2015		05/04/2015	
2015_2	87.50	synthetic	lambda-cyhalothrin 0.028 kg/ha 20/04/2015	tribenuron-methyl 0.04 kg/ha 20/05/2015	quizalofop-p 0.06 kg/ha 20/05/2015	31/05/2015	metaldehyde 0.05 kg/ha 20/05/2015
2015_3	92.00	synthetic		tribenuron-methyl 0.05 kg/ha 20/06/2015	quizalofop-p 0.0825 kg/ha 20/06/2015	30/06/2015	metaldehyde 0.05 kg/ha 15/05/2015
2015_5	32.00	synthetic		s-metolachlor 2.4 kg/ha 20/04/2015		15/05/2015	
2015_6	80.00	synthetic		imazamox 0.03 kg/ha 08/06/2015			metaldehyde 0.05 kg/ha 08/06/2015
2015_7	100.00	synthetic	lambda-cyhalothrin 0.01 kg/ha 01/04/2015	s-metolachlor 0.96 kg/ha 05/04/2015	pendimethalin 0.6825 kg/ha 05/04/2015		metaldehyde 0.05 kg/ha 05/04/2015
2015_8	0.00	none				01/07/2015	
2015_9	87.50	synthetic				12/06/2015	

B Pollination treatments

All pollination treatments were carried out during the flowering period of the crop. The exclusion treatment 'Pollinator-excluded' consisted of a whole sunflower head isolated from any insect visitation by means of a standard fibreglass mesh bag (mesh size 18x16 per in^2) placed before any floret opened (reproductive stages R3-R4 [1]) and removed when all stigmas withered (mean treatment length: 29 $\pm$ 4 days). For the 'Pollen supplemented' plants, the treatment consisted in repeatedly hand pollinating each plant using a paint brush to distribute fresh pollen collected from $>$ 20 donor plants; to ensure fresh and viable pollen all operations were carried out between 9:00 and 12:00 on four to five occasions, every other day following the sequential opening of the florets. 'Open pollination' plants were not manipulated in any way. All plants were labelled and collected individually when they reached physiological maturity (reproductive stage R9) [1]. Within each field, samples were taken at four distances (2, 16, 30, 44 m), according to the design described in the core text, and arranged as illustrated in Fig. B.

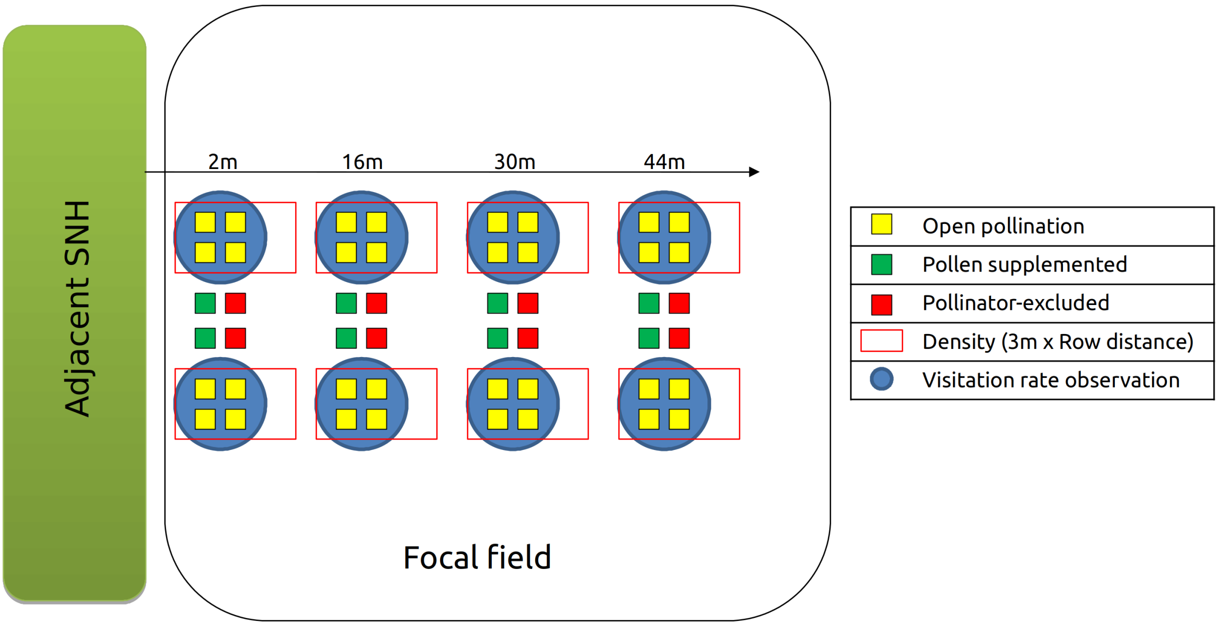

Fig B: **Schematic representation of the in-field sampling design (year 2014)** including the three treatments ('Open pollination', 'Pollen supplemented' and 'Pollinator-excluded') used to assess the degree of self-pollination, the actual level of pollination and the pollination deficit in sunflower commercial crops in the Pisa plain. Plants were chosen at 2, 16, 30, and 44 m from the adjacent focal SNHs. Plant density was assessed in two plots per distance (3m X distance among rows). Visitation rates were assessed on the same plants selected for the 'Open pollination' treatment.

C Land use characterisation

Table D: Description of land uses considered in this study.

Habitat	Description
Bare ground	Fields recently ploughed.
Cereals	Cereal crops, e.g. wheat (*Triticum aestivum* & associated spp), barley (*Hordeum sativum*), oats (*Avena sativa*), rye (*Secale cereale*).
Commercial horticulture	Nurseries.
Fruit trees	Fruit trees, e.g. apricots (*Prunus armeniaca*), peaches (*Prunus persica*), almonds (*Prunus dulcis*), citrus fruit (*Citrus* spp).
Grassy forage crops	Annually or biennially sown grass forages, e.g. *Phleum* spp, *Agropyron* spp, *Lolium* spp.
Legumes	Pulses and fodder crops (*Leguminosae*), e.g. alfalfa, clover, peas, lentils, lupins, beans.
Non habitat	Roads, lakes or any other habitat that do not fall into the rest of categories.
Olive groves	Olive (*Olea europea*).
Other annual crops	Annual herbaceous crops (except cereals and sunflower), e.g. maize (*Zea mays*), oilseed rape (*Brassica napus*).
Perennial herbaceous crops	Perennial herbaceous crops, e.g. asparagus (*Asparagus officinalis*), artichoke (*Cynara cardunculus*).
Rotational grassland	Rotational and interrupted grasslands (grasslands ploughed every 3-4 years and then sown with the same grass species).
Temporary in-field SNH (SNH FA)	Temporary in-field SNH: fallow, cover crops, not-marketable intercrops.
Herbaceous areal SNH (SNH HA)	Herbaceous areal elements: abandoned fields with less than 30% tree/shrub canopy cover or permanent and low-input grasslands.
Herbaceous linear SNH (SNH HL)	Herbaceous linear elements: any type of linear element (1.5 to 25 m wide) with less than 30% tree/shrub canopy cover.
Woody areal SNH (SNH WA)	Woody areal elements: Natural or semi-natural woodlands, including abandoned fields with more than 30% tree/shrub canopy cover.
Woody linear SNH (SNH WL)	Woody linear elements: any type of linear structure (1.5 to 25 m wide) with more than 30% tree/shrub canopy cover.
Sunflower	Sunflower (*Helianthus annuus*).
Turf	Leisure green areas, e.g. golf courses, football fields.
Urban	Urban areas.
Vegetable garden	Home orchards without commercial purposes.
Vineyards	Grape vine (*Vitis vinifera*).
Water course	Water courses > 1.5 m wide, e.g. rivers, streams, canals, drainage ditches.

Table E: Landscape sectors characteristics: main land uses. Year 2014. (n = 17)

Land use type	Mean area (%)	SD	Minimum	Maximum
Cereals	18.1	15.9	1.8	60.4
Sunflower	11.9	8.0	2.6	29.4
SNH WA	10.6	9.5	0.5	28.2
SNH HA	9.2	5.7	1.6	22.7
Urban	8.7	6.8	0.4	27.3
Legumes	6.0	6.1	0.0	19.1
Other annual crops	5.5	7.3	0.2	26.4
SNH HL	5.0	1.8	2.2	7.7
Grassy forage crops	4.9	4.5	0.0	15.7
SNH WL	2.8	1.4	0.2	5.2

D Wild bee species

Table F: Wild bee species list and their total abundance in 2014 and 2015. Note: The specimens that could not be captured for later identification are referred as 'Not identified Apoidea'.

Species	Functional group	2014	2015
Andrena (Zonandrena) flavipes	other wild bees	1	1
Bombus (Bombus) terrestris	bumble bees	7	26
Bombus (Melanobombus) lapidarius	bumble bees	11	5
Ceratina (Ceratina) cucurbitina	other wild bees	4	
Halictus (Halictus) quadricinctus	other wild bees		1
Halictus (Hexataenites) scabiosae	other wild bees	1	2
Lasioglossum (Dialictus) sp_2	other wild bees		1
Lasioglossum (Dialictus) politum	other wild bees	2	
Lasioglossum (Evylaeus) malachurum	other wild bees	1	
Lasioglossum (Evylaeus) sp_2	other wild bees	1	
Lasioglossum (Lasioglossum) discum	other wild bees	1	
Xylocopa (Xylocopa) violacea	other wild bees	8	
Not identified Apoidea	other wild bees	2	

E Pairwise comparisons of pollination treatments per cultivar

In order to test whether cross-pollination affected both response variables for each and every one of the cultivars, pairwise comparisons of pollination treatments per cultivar were performed. Hand pollinated plants had significantly higher levels of seed set than bagged ones for all cultivars. For oil content, differences between treatments were also significant for all cultivars except one: *Mas 86.OL-Maisadour*. Year was not significant either for seed set ($\chi^2 = 0.204$, df = 1, $p = 0.65$) or oil content ($\chi^2 = 2.886$, df = 1, $p = 0.09$) and thus was removed from the minimal adequate models (table G).

Table G: Pairwise comparison of pollination treatments per cultivar for seed set and oil content. Note: *** p < 0.001, ** p < 0.01, * p < 0.05.

		Seed set			Oil content		
Cultivar	Contrast	z ratio	p value	Significance	z ratio	p value	Significance
Heliawin-KWS	Poll-excl − Poll suppl	-7.684	0.000	* * *	-3.124	0.002	* *
Imeria-Caussade	Poll-excl − Poll suppl	-4.259	0.000	* * *	-2.022	0.043	*
Inostarck-Apsov	Poll-excl − Poll suppl	-9.040	0.000	* * *	-4.422	0.000	* * *
Inotop-Apsov	Poll-excl − Poll suppl	-4.812	0.000	* * *	-3.885	0.000	* * *
Klarika Cl-Caussade	Poll-excl − Poll suppl	-7.606	0.000	* * *	-3.059	0.002	* *
LG 55.57 HO-LG	Poll-excl − Poll suppl	-9.562	0.000	* * *	-7.424	0.000	* * *
LG 56.56 HO-LG	Poll-excl − Poll suppl	-8.545	0.000	* * *	-5.710	0.000	* * *
Mas 83.R-Maisadour	Poll-excl − Poll suppl	-7.175	0.000	* * *	-3.873	0.000	* * *
Mas 86.OL-Maisadour	Poll-excl − Poll suppl	-2.416	0.016	*	-1.544	0.123	
P64HE39-Pioneer	Poll-excl − Poll suppl	-6.870	0.000	* * *	-2.982	0.003	* *
PR64H41-Pioneer	Poll-excl − Poll suppl	-4.893	0.000	* * *	-2.626	0.009	* *
PR64H42-Pioneer	Poll-excl − Poll suppl	-5.546	0.000	* * *	-2.570	0.010	*
Sangria CS-Caussade	Poll-excl − Poll suppl	-7.475	0.000	* * *	-4.028	0.000	* * *

F Effect of visitation rates: models outputs

Table H: Effect of visitation rates and CPD on seed set and oil content of sunflower: model estimates and standard errors (listed in parentheses). Notes: Empty coefficients stand for parameters not included in the model; 'Visitation rates' were log transformed.

	Model 1: Seed set	Model 2: Oil content
(Intercept)	2.15 (0.42)***	0.13 (0.06)*
Visitation rates	-0.02 (0.03)	0.01 (0.00)
CPD Seed set	-3.33 (0.75)***	
Visitation rates : CPD Seed set	1.00 (0.06)***	
CPD Oil content		-1.30 (0.78)
Visitation rates : CPD Oil content		0.71 (0.05)***
BIC	-1592.20	-3311.36
Log Likelihood	819.46	1679.04
Num. obs.	792	792
Num. groups: field	25	25
Num. groups: field:plot	83	83

$^{***}p < 0.001$, $^{**}p < 0.01$, $^{*}p < 0.05$

G Landscape drivers of pollination service delivery

G.A Correlation between explanatory variables

Pearson's correlation coefficients between all landscape explanatory variables are reported in table I. Combinations between significantly correlated variables (Pearson's correlation coefficient r > 0.5, $p < 0.05$) were excluded when performing automated model selection.

Table I: Pearson's correlation matrix of landscape variables. Significantly collinear variables are bold formatted. 'CPD Seed set': Cross-pollination dependence of each cultivar for seed set; 'Beehives hmp': Heatmap value derived from the number of beehives in 1.5 km; 'Gr. forage cr.': Grassy forage crops; 'Oth. annual cr.': Other annual crops. Note: *** p < 0.001, ** p < 0.01, * p < 0.05.

	CPD Seed set	Beehives hmp	Cereals	Gr. forage cr.	Legumes	Oth. annual cr.	SNH HA	SNH HL	SNH WA	SNH WL	Sunflower
CPD Seed set											
Beehives hmp	0.21										
Cereals	0.04	0.04									
Gr. forage cr.	0.39	-0.13	-0.03								
Legumes	0.15	0.06	0.01	-0.29							
Oth. annual cr.	-0.17	-0.28	-0.23	-0.32	0.03						
SNH HA	-0.04	-0.21	-0.39	0.15	-0.33	-0.46					
SNH HL	-0.14	0.29	-0.20	-0.02	0.43	-0.08	-0.38				
SNH WA	0.03	0.15	**-0.52***	-0.13	0.09	-0.31	**0.54***	0.02			
SNH WL	0.04	-0.09	-0.11	-0.06	0.17	-0.36	0.45	0.06	0.33		
Sunflower	-0.08	0.24	0.08	0.36	-0.45	0.14	-0.37	0.00	-0.28	**-0.58***	
Urban	0.10	-0.16	-0.22	-0.13	0.15	-0.17	0.30	0.20	-0.09	0.40	-0.49

G.B Relevant landscape variables for pollination service

To identify the most important landscape variables affecting pollination service delivery we fitted every possible model except those ones with collinear predictors (section section G.A) using the `dredge` function (MuMIn package [2]) (table K). We considered as best models those ones with $\delta < 5$, being δ the difference in AICc between each model and the best model. Then, for the subset of these best models, we calculated the relative importance of each individual parameter as the sum of 'Akaike weights' (normalized model likelihoods) over all models including that parameter, and considered as potentially good candidates all those landscape variables with a relative importance > 0.2 (table J).

Table J: Relative importance of landscape variables. Relative importance of each parameter is the sum of the normalized likelihoods of the models with $\delta < 5$ that include that parameter. 'Beehives hmp': Heatmap value derived from the number of beehives in 1.5 km. Note: local variables as 'Adjacent SNH', 'Distance' and 'CPD Seed set' of the cultivar were coerced to be present in all models as part of the two step selection procedure.

	Importance	N containing models
Adjacent SNH	1.00	8
Beehives hmp	1.00	8
Distance	1.00	8
CPD Seed set	1.00	8
SNH HA	1.00	8
SNH HL	1.00	8
SNH WL	1.00	8
Urban	1.00	8
Other annual crops	0.88	6
Legumes	0.30	3
Cereals	0.17	3
Grassy forage crops	0.12	2
SNH WA	0.00	0
Sunflower	0.00	0

Table K: Models explaining pollination service according to AICc. Best models with $\delta < 5$ are grey shaded. Adjacent SNH, distance and cross-pollination dependence of each cultivar were coerced to be present in all models as covariates. 'Adj. SNH': Adjacent SNH; 'Beehives hmp': Heatmap value derived from the number of beehives in 1.5 km; 'Dist.': Distance; 'Gr. forage cr.': Grassy forage crops; 'Oth. annual cr.': Other annual crops; 'CPD SS': Cross-pollination dependence of each cultivar for seed set.

Rank	(Intercept)	Adj. SNH	Beehives hmp	Cereals	Dist.	Gr. forage cr.	Legumes	Oth. annual cr.	CPD SS	SNH HA	SNH HL	SNH WA	SNH WL	Sunflower	Urban	df	logLik	AICc	delta	weight
1	-2.093	+	0.005		+			1.084	4.030	-3.005	-8.053		11.060		2.088	15	141.813	-244.395	0.000	0.336
2	-2.169	+	0.005		+		-0.571	1.184	4.139	-3.195	-7.715		12.608		2.045	16	142.921	-243.175	1.220	0.182
3	-2.104	+	0.005		+	0.284		1.159	3.994	-3.041	-8.205		11.400		2.116	16	141.914	-241.161	3.234	0.067
4	-2.150	+	0.005	0.063	+			1.192	4.041	-2.806	-7.656		10.881		2.091	16	141.847	-241.027	3.368	0.062
5	-1.774	+	0.004		+				3.858	-3.715	-7.882		8.995		2.138	14	138.253	-240.581	3.814	0.050
6	-1.640	+	0.004	-0.335	+				3.896	-4.397	-10.077		11.019		2.096	15	139.799	-240.367	4.028	0.045
7	-2.169	+	0.005		+	-0.138	-0.608	1.154	4.164	-3.189	-7.619		12.545		2.028	17	142.943	-239.646	4.749	0.031
8	-2.131	+	0.005	-0.046	+		-0.593	1.110	4.136	-3.345	-7.986		12.798		2.041	17	142.939	-239.638	4.757	0.031
9	-1.657	+	0.004	-0.428	+		-0.689		4.018	-4.901	-10.285		13.226		2.043	16	140.945	-239.223	5.172	0.025
10	-2.897	+	0.005	0.715	+			2.389	4.154				7.477		1.711	14	137.363	-238.801	5.594	0.020
11	-1.801	+	0.004		+		-0.351		3.914	-3.878	-7.692		9.834		2.118	15	138.515	-237.799	6.596	0.012
12	-1.792	+	0.004		+	-0.495			3.941	-3.570	-7.643		8.641		2.087	15	138.498	-237.765	6.630	0.012
13	-2.152	+	0.005	0.054	+	0.272		1.248	4.005	-2.870	-7.860		11.232		2.117	17	141.939	-237.638	6.757	0.011
14	-1.644	+	0.004	-0.329	+	-0.052			3.904	-4.370	-10.017		10.950		2.092	16	139.802	-236.937	7.458	0.008
15	-2.881	+	0.006	0.701	+			2.546	4.196		-2.256		8.363		1.908	15	138.029	-236.827	7.568	0.008
16	-2.729	+	0.004		+			1.679	4.303			-1.119	12.410			13	134.559	-236.377	8.018	0.006
17	-1.703	+	0.003	-0.391	+	-0.531	-0.826		4.126	-4.730	-9.706		12.957		1.981	17	141.226	-236.212	8.183	0.006
18	-2.759	+	0.005	0.595	+			2.045	4.104	-0.559			7.555		1.652	15	137.623	-236.015	8.380	0.005
19	-2.129	+	0.004	-0.048	+	-0.144	-0.633	1.075	4.161	-3.348	-7.901		12.742		2.023	18	142.963	-235.967	8.428	0.005
20	-1.858	+	0.004		+	-0.928	-0.644		4.119	-3.741	-7.080		9.871		2.004	16	139.230	-235.793	8.602	0.005
21	-2.935	+	0.005	0.719	+		-0.197	2.465	4.199				8.038		1.705	15	137.448	-235.665	8.730	0.004
22	-2.885	+	0.005	0.712	+	-0.162		2.341	4.174				7.314		1.703	15	137.383	-235.535	8.860	0.004
23	-2.664	+	0.004		+	-0.951		1.399	4.420			-1.156	11.478			14	135.073	-234.221	10.174	0.002
24	-2.559	+	0.006	0.709	+			1.809	3.964						2.260	13	133.451	-234.161	10.234	0.002
25	-2.048	+	0.003		+				3.834	-2.565			5.865		1.275	13	133.410	-234.079	10.316	0.002
26	-2.714	+	0.004		+			1.698	4.259			-1.010	11.028		0.465	14	134.964	-234.003	10.392	0.002
27	-2.339	+	0.004		+			0.980	3.993	-1.923			7.654		1.214	14	134.917	-233.909	10.486	0.002
28	-1.372	+	0.006		+				3.722	-3.409	-7.567			-1.105	2.412	14	134.908	-233.891	10.504	0.002
29	-2.725	+	0.004		+			1.791	4.346		-1.594	-1.155	13.480			14	134.836	-233.747	10.648	0.002
30	-2.892	+	0.006	0.703	+		-0.053	2.562	4.207		-2.196		8.491		1.901	16	138.035	-233.403	10.992	0.001
31	-2.877	+	0.006	0.700	+	-0.052		2.529	4.202		-2.241		8.306		1.904	16	138.031	-233.395	11.000	0.001
32	-2.769	+	0.004		+		-0.215	1.758	4.352			-1.124	13.005			14	134.632	-233.339	11.056	0.001
33	-2.765	+	0.005	0.534	+		-0.442	2.015	4.176	-0.884			8.860		1.605	16	137.978	-233.289	11.106	0.001
34	-1.904	+	0.004		+				3.748	-2.169					1.734	12	131.451	-233.229	11.166	0.001
35	-1.699	+	0.004		+				3.735	-2.717	-4.609				2.383	13	132.710	-232.679	11.716	0.001
36	-2.749	+	0.005	0.593	+	-0.154		2.000	4.123	-0.556			7.400		1.645	16	137.641	-232.615	11.780	0.001
37	-2.509	+	0.006	0.755	+			1.969	3.982					-0.595	2.086	14	134.269	-232.613	11.782	0.001
38	-2.425	+	0.003		+		-0.778	1.118	4.141	-2.250			9.936		1.211	15	135.820	-232.409	11.986	0.001
39	-2.926	+	0.005	0.714	+	-0.350	-0.280	2.392	4.262				7.924		1.685	16	137.528	-232.389	12.006	0.001
40	-2.112	+			+				4.087	-2.619			9.317			11	129.551	-232.388	12.008	0.001
...																				
959	-1.983	+	0.004	0.240	+	-1.067	-0.669	0.625	4.193	-1.269	1.101			-1.113		17	129.355	-212.470	31.925	0.000
960	-1.635	+		-0.129	+	-2.077	-0.770	-0.751	4.304	-3.074	-1.276			-0.233	1.361	17	129.222	-212.204	32.191	0.000

G.C Model outputs

Table L: Effect of local and landscape variables on pollination service delivery: model estimates and standard errors (listed in parentheses).

	Pollination service
(Intercept)	-2.078 (0.157)***
CPD Seed set	4.030 (0.155)***
Adj. Grassy SNH	0.037 (0.067)
Adj. Woody SNH	-0.270 (0.055)***
Beehives heatmap	0.005 (0.001)***
Proportion of HA	-2.990 (0.493)***
Proportion of HL	-8.031 (1.732)***
Proportion of WL	11.051 (1.944)***
Proportion of urban areas	2.088 (0.369)***
Proportion of other annual crops	1.091 (0.356)**
BIC	-230.672
Log Likelihood	140.653
Num. obs.	68
Num. groups: field	17

$^{***}p < 0.001$, $^{**}p < 0.01$, $^{*}p < 0.05$

References

[1] A A Schneiter and J F Miller. Description of Sunflower Growth Stages. *Crop Science*, 21:901–903, 1981. doi: 10.2135/cropsci1981.0011183X002100060024x. URL http://dx.doi.org/10.2135/cropsci1981.0011183X002100060024x.

[2] Kamil Barton. MuMIn: Multi-Model Inference, 2016. URL https://cran.r-project.org/package=MuMIn.
